# Aromatic profiles and 15-LOX inhibitory effects of Australian and New Zealand Manuka honeys

**DOI:** 10.1371/journal.pone.0361877

**Published:** 2026-11-03

**Authors:** Georgia Moore, Asmaa Boufridi, Fraser Russell, Peter Brooks

**Affiliations:** 1 School of Science, Technology and Engineering, University of the Sunshine Coast, Maroochydore BC, Queensland, Australia; 2 Centre for Bioinnovation, School of Science, Technology and Engineering, University of the Sunshine Coast, Maroochydore BC, Queensland, Australia; 3 School of Health, University of the Sunshine Coast, Maroochydore BC, Queensland, Australia; Cairo University, EGYPT

## Abstract

Manuka (*Leptospermum*) honeys from Australia (Aust) and New Zealand (NZ) contain high levels of aromatic compounds, though only a subset are true phenolics in that they contain a hydroxyl group attached to an aromatic ring. Consequently, the traditional Folin-Ciocalteu Total Phenolic Content (FC-TPC) assay fails to detect many non-phenolic aromatic compounds that may confer health benefits. This paper quantified both phenolic and non-phenolic aromatic compounds in monofloral manuka honeys using high-performance liquid chromatography with diode array detection (HPLC-DAD) and the FC-TPC assay. In addition, this study evaluated the capacity of the honeys to inhibit 15-lipoxygenase (15-LOX), an enzyme that causes peroxidation of long chain polyunsaturated fatty acids. Australian (n = 7) and New Zealand (n = 6) *Leptospermum scoparium* (*L. scoparium*) honeys showed the highest total aromatic concentrations by HPLC-DAD (>2100 mg/kg), significantly exceeding those of *L. liversidgei* (n = 5), *L. whitei* (n = 5), and *L. speciosum* (n = 3) honeys. The FC-TPC assay identified higher phenolic levels in *L. scoparium* and *L. polygalifolium* honeys than in the other species studied. Sugar free Australian and New Zealand manuka honey aromatic extracts were potent inhibitors of 15-LOX enzyme, with IC_50_ values recorded at <15% of stock solutions (total extractables from 20.0 g of honey dissolved in 1.0 mL of 50% aqueous ethanol). Australian (IC_50_, 13.2 ± 0.7%) and New Zealand (13.1 ± 1.9%) *L. scoparium* honeys were more potent in inhibiting 15-LOX than *L. liversidgei* (IC_50_, 37.5 ± 6.3%) and *L. speciosum* (IC_50_, 36.6 ± 10.5%). Overall, this study demonstrates that HPLC-DAD provides a more comprehensive assessment of aromatic compounds in manuka honey than the FC-TPC assay and highlights the potential contribution of these compounds to anti-inflammatory activity.

## Introduction

Manuka honey is produced by honeybees (*Apis mellifera*) that forage on the nectar of *Leptospermum* (Myrtaceae) plants in Australia and New Zealand [1]. Mounting evidence from *in vitro* and, to a lesser extent, *in vivo* studies demonstrates that manuka honey possesses anti-inflammatory, anti-cancer, antidiabetic and antioxidant activity [2–9]. It has also been shown to reduce pathology in rat models with experimentally induced gastric ulcers and inflammatory bowel disease [2,10] and improve outcomes in patients with gastro-esophageal reflux disease (GERD) [11].

Manuka honey is best known for its non-peroxide antibacterial activity (NPA), due to the presence of methylglyoxal (MGO), a compound exclusive to this honey type [12,13]. This antibacterial activity is strongly linked to its effectiveness in wound care [14–17]. Manuka honey disrupts established bacterial biofilms [18], a key factor in the chronicity of wound infection, thereby enhancing antimicrobial action and promoting wound healing [16,19]. Clinically, non-healing wounds in patients treated with high-MGO manuka honey (850 + mg/kg) have shown complete wound size reduction and new tissue growth within four weeks [20]. While another study reported reduced bacterial colonisation and shortened hospital stays in patients with diabetic foot ulcers treated with manuka honey compared to conventional dressings [21]. Within this context, the antibacterial activity of manuka honey and its association with MGO is well established. However, the mechanisms underlying its antioxidant and anti-inflammatory effects are more complex, depending not only on the presence of bioactive constituents, but also their chemical nature and interactions within the honey matrix [19,22].

The biological activity of manuka honey is underpinned by its chemically complex matrix, composed primarily of sugars and water (~95%), with amino acids, proteins, enzymes, organic acids, vitamins, aromatic (phenolic and non-phenolic) compounds, minerals and volatile compounds comprising the remaining 2–5% [23]. Although present in minor quantities, aromatic compounds substantially influence the organoleptic and biological properties of honey [24]. Aromatic compounds can be broadly classified into phenolic and non-phenolic groups [25]. Phenolic compounds contain hydroxylated aromatic rings that confer antioxidant bioactivity [23,26,27], whereas non-phenolic aromatic compounds lack these hydroxyl groups on the aromatic ring [27], but still represent a diverse and abundant class of radical scavengers with biological activity [28,29].

The aromatic profile of *L. scoparium* honey has been extensively characterised; however, this species represents only one source of manuka honey. *Leptospermum polygalifolium* is the second most studied species; although only a limited number of studies have reported its chemical profile, two of which were based on a single sample (*n* = 1) [30–32]. To date, no published information is available on the aromatic profiles of manuka honeys derived from *L. whitei*, *L. liversidgei*, and *L. speciosum* plant species*.* Manuka honey contains a range of phenolic compounds, flavonoids and other aromatic constituents. Leptosperin, a nectar-derived glycoside of methyl syringate, is one of the most characteristic and well-known compounds of manuka honey, having been identified in all *leptospermum* varietals investigated [3,33,34]. Other reported compounds include 4-methoxyphenyllactic acid, lepteridine, 2-methoxybenzoic acid, syringic acid, 3-phenyllactic acid, 3,4,5-trimethoxybenzoic acid, phenylacetic acid, gallic acid, and flavonoids such as, pinocembrin, chrysin, pinobanksin, and luteolin [34].

The aromatic composition of honey varies widely with botanical origin, geographic region, seasonal conditions and methods of analysis. Nevertheless, manuka honey derived from *L. scoparium* has been noted to contain high levels of phenolic compounds (>2400 mg/kg gallic acid equivalents; GAE), as determined using the FC-TPC method [35]. The FC-TPC method, while commonly used, selectively detects phenolic aromatic compounds based on their reducing capacity, but may overlook other biologically active non-phenolic aromatic compounds [26], thereby limiting its suitability as a stand-alone measure. HPLC-DAD provides a complementary analytical approach by enabling the identification and quantification of individual aromatic constituents, including compounds that may not be detected by the FC-TPC assay.

Importantly, several non-phenolic aromatic compounds including lepteridine, leptosperin, 3-phenyllactic acid, 2-methoxyacetophenone, 2-methoxybenzoic acid and 4-methoxyphenyllactic acid, are major constituents of manuka honey and may contribute to its anti-inflammatory and antioxidant profiles but are not detected by the standard FC-TPC assay. Notably, significant concentrations of 3-phenyllactic acid, 4-methoxyphenyllactic acid, leptosperin, and methyl syringate remain present after *in vitro* simulated digestion of manuka honey [36]. In contrast, MGO is largely metabolised in the gastrointestinal tract [37]. This suggests that aromatic compounds may contribute more to the bioactivity of manuka honey than previously recognised, despite the prevailing emphasis on MGO as its principal active constituent. Consistent with this, higher MGO content does not necessarily correspond to greater antioxidant potential. For example, a manuka honey containing 250 mg/kg MGO has been shown to exhibit higher antioxidant potential than one containing 400 mg/kg [38,39].

A growing body of evidence indicates that manuka honey can dampen oxidative stress and inflammatory pathways [2,5,40]. Several aromatic compounds in manuka honey can scavenge reactive oxygen species (ROS) and reduce nitrate accumulation [5,41]. Leptosperin for example, is primarily hydrolysed to methyl syringate upon ingestion [3,42], and methyl syringate exerts antioxidant and anti-inflammatory effects by inhibiting myeloperoxidase (MPO) activity [43] and reducing neutrophil production of ROS [41]. Syringic acid has been associated with hepatoprotective effects, hypoglycaemic properties, anti-inflammatory effects and anti-microbial activity [23].

Additionally, lepteridine, a heteroaromatic compound that is unique to manuka honey, is suggested to partially inhibit the activity of matrix metalloproteinase-9 (MMP-9) by interfering with its ability to degrade gelatine and extracellular matrix proteins [44]. MMP-9 is upregulated in non-healing wounds and inflammatory bowel disease [45,46]. Lin, Nair [44] demonstrated that lepteridine remains largely stable during simulated *in vitro* digestion of manuka honey, indicating potential bioavailability after oral consumption. Manuka honey has also been shown to suppress the expression of inflammatory mediators, including tumour necrosis factor-alpha (TNF-α), interleukin (IL)-1β and IL-6, and inducible nitric oxide synthase (iNOS) [5].

Beyond these mechanisms, modulation of oxidative lipid-mediator pathways, particularly those involving 15-LOX may represent another pathway through which manuka honey exerts its biological effects. The enzyme 15-LOX, encoded by the human ALOX15 gene, catalyses the conversion of polyunsaturated fatty acids (PUFAs) that contains a 1-cis, 4-cis pentadiene structure to hydroperoxy products. 15-LOX is expressed in eosinophils, macrophages, reticulocytes, bronchial epithelial cells and skin [47], and catalyses the peroxidation of linoleic acid, arachidonic acid and docosahexaenoic acid to 13-hydroperoxyoctadecadienoic acid, 15-hydroperoxyeicosatetraenoic acid and 17-hydroxydocosahexaenoic acid, respectively.

The peroxidation of PUFAs is causally linked to altered plasma membrane structure and function and to the propagation of ROS, culminating in damage to cell DNA, lipids and proteins [48]. While these injurious effects have been hypothesised to contribute to diabetes mellitus, endothelial dysfunction and atherosclerosis [49,50], 15-LOX activity may also contribute to the termination of inflammation by catalysing the formation of pro-resolving mediator precursors [51]. Noting this “double-edged” role of 15-LOX, suppression of 15-LOX activity may be beneficial to human health under specific circumstances, for example where enzyme activity has become dysregulated. While inhibitory effects of polyphenols from green and black tea on lipoxygenase activity have been described previously [52], the efficacy of aromatic extracts of manuka honeys is less well known.

Limited information is available regarding the aromatic profiles of honeys derived from different *Leptospermum* varieties, particularly Australian species, and whether variations in these profiles can modify 15-LOX activity. Characterising the aromatic profile of manuka honey and assessing its capacity to inhibit 15-LOX may provide important mechanistic insights into its potential to protect against lipid peroxidation. In this paper we aim to; (1) identify the aromatic profile of different *Leptospermum* derived manuka honeys, (2) compare the total aromatic content using HPLC-DAD analysis and the total phenolic content using the FC-TPC assay, and (3) investigate the potency of sugar-free aromatic extracts of manuka honeys for inhibiting soybean 15-LOX activity using a cell-free assay.

## Materials and methods

### Chemicals and reagents for HPLC-DAD and leptosperin analysis

All HPLC-grade solvents used for extraction and chromatography, including acetonitrile (ACN), methanol (MeOH) and ethanol (EtOH), were sourced from Merck Australia (Victoria, Australia). Ultrapure water (MilliQ) was obtained from a Milli-Q® Direct Water Purification System (18.2 MΩ·cm). Analytical standards were obtained from Fluka Chemicals (Castle Hill, Australia), Sigma-Aldrich (Castle Hill, Australia) or collaborators from the University of Western Australia and Auckland University (S1 Table).

### Honey samples

Manuka honey samples (MGO 263–2003 mg/kg) from Australia (five *Leptospermum* species, total *n =* 26) and New Zealand (one *Leptospermum* species, *n =* 6) were selected for their high MGO content and analysed in this study to determine their aromatic profiles (Table 1). Australian manuka honey was supplied by Tyagarah Apiaries (Australia’s Manuka Pty Ltd), Australian Jelly Bush Pty Ltd, King Island Pure Pty Ltd and Daybreak Apiaries Pty Ltd. These include *L. scoparium* (*n* = 7) from Tasmania; *L. polygalifolium* (*n* = 6), *L. liversidgei* (*n* = 5), *L. whitei* (*n* = 5) and *L. speciosum* (*n* = 3), all from New South Wales. Queensland Grey Ironbark (*Eucalyptus paniculata*), Western Australian Jarrah (*Eucalyptus marginata*) and Queensland Brush Box (*Lophostemon confertus*) honey were used as non-manuka comparators in the 15-LOX inhibition assay. The six New Zealand *L. scoparium* honey samples were purchased in February 2023 from Chemist Warehouse pharmacy stores in Queensland (Comvita®) or provided by New Zealand Honey Export Group Limited. Additional details on the geographical origin and extraction of the honeys included in this study are provided in Table 1. Honeys were stored in the dark at 4 °C prior to analysis to minimise changes in their chemical profiles [13,53]. Before sampling, honey samples were brought to room temperature (22 °C) and mixed thoroughly by stirring.

**Table 1:** Sample details for Australian and New Zealand monofloral manuka honeys analysed (n = 32).

Sample ID	DHA (ppm)	MGO (ppm)	Floral Source	Geographical Origin (postcode)	Collection Year
**LL1**	238	263	*L. liversidgei*	2481	2019
**LL2**	934	335	*L. liversidgei*	2477	2024
**LL3**	2044	1603	*L. liversidgei*	2472	2019
**LL4**	1916	1558	*L. liversidgei*	2472	2019
**LL5**	1423	1109	*L. liversidgei*	2472	2019
**LP1**	1370	839	*L. polygalifolium*	2481	2019
**LP2**	3313	652	*L. polygalifolium*	2472	2020
**LP3**	1602	1252	*L. polygalifolium*	2472	2019
**LP4**	4660	1243	*L. polygalifolium*	2472	2021
**LP5**	1925	1150	*L. polygalifolium*	2472	2019
**LP6**	1739	2003	*L. polygalifolium*	NA	2020
**LW1**	1498	465	*L. whitei*	2478	2021
**LW2**	1373	961	*L. whitei*	2472	2019
**LW3**	1795	473	*L. whitei*	2481	2020
**LW4**	1999	656	*L. whitei*	2472	2019
**LW5**	1487	1074	*L. whitei*	2473	2020
**LS1**	669	537	*L. scoparium* (Aust)	7321	2019
**LS2**	1391	590	*L. scoparium* (Aust)	7256	2020
**LS3**	3680	415	*L. scoparium* (Aust)	7256	2021
**LS4**	1270	762	*L. scoparium* (Aust)	7256	2022
**LS5**	1640	1085	*L. scoparium* (Aust)	7256	2021
**LS6**	3468	435	*L. scoparium* (Aust)	7256	2021
**LS7**	2308	439	*L. scoparium* (Aust)	7256	2020
**LSp1**	1502	442	*L. speciosum*	2473	2020
**LSp2**	1358	407	*L. speciosum*	2473	2020
**LSp3**	2628	279	*L. speciosum*	2473	2022
**NZ1**	483	403	*L. scoparium* (NZ)	NA	NA
**NZ2**	655	658	*L. scoparium* (NZ)	NA	NA
**NZ3**	1039	1145	*L. scoparium* (NZ)	NA	NA
**NZ4**	794	1060	*L. scoparium* (NZ)	NA	2020
**NZ5**	530	960	*L. scoparium* (NZ)	NA	2020
**NZ6**	1632	1296	*L. scoparium* (NZ)	NA	2020

NA = Not Available.

### HPLC-DAD analysis of dihydroxyacetone and methylglyoxal

The dihydroxyacetone (DHA) and MGO content of all honeys were derivatised using O-2,3,4,5,6-(pentafluorobenzyl)hydroxyl amine hydrochloride (PFBHA), then quantified by HPLC-DAD array, based on the method established by Pappalardo, Pappalardo [54].

### Fluorescence analysis of leptosperin

The leptosperin content of honeys was determined using fluorescence analysis Brooks, Boufridi [55]. Briefly, the honey sample was diluted to a concentration of 1.0 g/5 mL (20% m/v). A 0.5 mL aliquot of each sample was then diluted with 9.5 mL absolute EtOH to achieve a final concentration of 0.01 g/mL (1% m/v). Samples (300 μL) were analysed at _ex_270nm - _em_365nm using a Perkin Elmer Enspire Multimode Plate Reader with a xenon light source.

### HPLC-DAD analysis of aromatic compounds in non-concentrated honey extracts

Honeys were dissolved in MilliQ water to a concentration of 20% m/v and analysed directly by HPLC-DAD as described by Moore, Brooks [56]. Briefly, standards (100 µg/mL) and samples were analysed by HPLC–DAD, using an Agilent 1290 Infinity II HPLC equipped with a Phenomenex Synergi Fusion-RP column (150 × 4.6 mm, 4 µm) and coupled to an Agilent 1260 Infinity DAD with multiwavelength detection (200–330 nm). The mobile phase comprised water–acetonitrile (98:2, v/v) with 0.004% TFA (MPA) and 100% acetonitrile (MPB). Compounds were identified by comparing retention time and UV/Vis spectra with our in-house library of 110 authentic standards. Aromatics compounds were quantified using calibration curves (R^2^ > 0.9963) [56] and expressed as mg/kg honey. Results are reported as mean ± SEM.

### Folin-Ciocalteu Total Phenolic Content (FC-TPC) assay

The total phenolic assay was modified from Lawag, Nolden [57]. Folin–Ciocalteu 2 N solution and sodium carbonate were purchased from Sigma Aldrich (Castle Hill, Australia). Honey samples were dissolved in MilliQ water to a final concentration of 1.0 g/2.5 mL. A stock solution of gallic acid was prepared at a concentration of 12.5 mg/ mL in 100% MeOH. Four-fold serial dilutions of the gallic acid standard and honey solutions were prepared using MeOH diluent. Folin-Ciocalteu solution (80 µL) was added to 400 µL of MilliQ water, followed by 20 µL of diluted honey solution or gallic acid standard solution in MeOH. Sodium carbonate (1% m/v; 500 µL) was added and the mixture was incubated for 60 min in darkness at room temperature. MeOH 100% was used as a reagent blank. Samples, standards and reagent blank were plated in a 96-well plate (200 µL per well in triplicate) and absorbance was measured at 760 nm using a Perkin Elmer EnSpire Multimode Plate Reader. Total phenolic content was expressed as gallic acid equivalents (GAE) in mg/kg. Results are reported as mean ± SEM.

### Preparation of concentrated honey extracts for 15-LOX inhibition assay

Honey (20 g) was dissolved in 15 mL of warm MeOH (40 °C) and vigorously shaken to ensure complete dissolution. Separately, 150 mL of ACN was pre-warmed to 45 °C in a conical flask and maintained at temperature in a 45 °C bath. The dissolved honey solution was added to the ACN and stirred for 3–5 minutes using a magnetic stirrer. This solution was transferred to a freezer and left at −20 °C overnight (~18 h) to allow the sugars to precipitate. After freezing, the supernatant was collected and concentrated using a rotary evaporator with a warm water bath (35 °C).

An Oasis HLB 12cc 500 mg LP extraction cartridge (Waters^TM^), was prepared for solid phase extraction (SPE) as follows: the cartridge was first rinsed with four x 1 mL aliquots of ACN and then conditioned with three x 1 mL aliquots of MPA. The dried aromatic extract was recovered by washing the flask with three consecutive aliquots (1 mL each) of MPA. The three aliquots were sequentially loaded onto the SPE cartridge. The cartridge was washed with three aliquots of MilliQ water (1 mL), and gentle air pressure from a syringe was used to release residual liquid. The sample was eluted from the cartridge using four x 1 mL aliquots of ACN and collected into a test tube. An orange aromatic band was visually observed during elution.

The eluate was transferred to a pre-weighed round bottom flask and concentrated using a rotary evaporator in a warm water bath maintained at 35 °C. The dried residue typically weighing 20–40 mg was reconstituted with 1 mL of 50:50 (v/v) EtOH: MilliQ water solution for the 15-LOX assay. This extract representing 20.0 g of honey per 1.0 mL is referred to as the stock aromatic solution during this assay.

### 15-Lipoxygenase (15-LOX) assay

The 15-LOX assay was modified from Anthon and Barrett [58]. A serial dilution of the stock aromatic solution (100%) was used to prepare 85.0%, 70.0%, 55.0%, 40.0%, 20.0%, 5.0%, 3.8% and 1.5% intermediate solutions. A 0.4 M solution of 3-(dimethylamino)benzoic acid (DMAB, Sigma-Aldrich), prepared in 1 M HCl (2.5 mL), was diluted with 0.1 M sodium phosphate solution, pH 6.0, to form a 20 mM solution (50 mL). Solution A was prepared by diluting the 20 mM DMAB to a final concentration of 10 mM using MilliQ water diluent. Aliquots (50 µL) of stock and intermediate aromatic extracts were added to 222 µL of Solution A.

Linoleic acid (Sigma Aldrich, 5.0 µL of 25 mM stock that was prepared in MilliQ water containing Tween-20 and clarified with sodium hydroxide) and 15-LOX enzyme (653,000 units/mg protein of soybean lipoxidase, Sigma-Aldrich; 1.0 µL) were added to samples and incubated for 5 min at 21°C. Controls included i) substitution of an equivalent volume of ethanol/water mixture for the aromatic extract (to define maximum 15-LOX activity); ii) substitution of an equivalent volume of MilliQ water for the linoleic acid; and iii) substitution of an equivalent volume of MilliQ water for the 15-LOX enzyme.

Solution B (222 µL), containing 0.2 mM 3-methyl-2-benzothiazolinone hydrazone hydrochloride hydrate (Sigma-Aldrich) and 0.1 mg/mL bovine blood haemoglobin (Sigma-Aldrich) in MilliQ water, was added to each sample and incubated for a further 5 min at 21°C. Duplicate 200 µL aliquots of each sample were dispensed into a 96 well plate and read at 590 nm using an EnSpire Multimode Plate Reader (PerkinElmer).

Absorbance was plotted against the percent concentration of aromatic extract and an IC_50_ value was determined using the second-order polynomial equation for the line of best fit. Absorbance was plotted against the concentration of aromatic extract, and IC_50_ values were determined using a second-order polynomial fit, with IC_50_ defined as the concentration achieving 50% of the y-intercept.

### Statistical analysis

Statistical analyses were performed using IBM SPSS Statistics (Version 29.0, Build 241). Data were tested for normality using the Shapiro–Wilk test (P > 0.05), with means and standard deviations reported for normally distributed data. Differences in 15-LOX inhibitory potency and concentration of aromatics in *Leptospermum* species were evaluated using one-way ANOVA, followed by Tukey’s HSD post hoc test. Differences were considered statistically significant at p < 0.05. IC_50_ values are expressed as a percentage of the original aromatic concentration.

## Results and discussion

### Dihydroxyacetone and methylglyoxal content

The honeys selected all displayed MGO levels of ≥ 263 mg/kg (Australian *L. scoparium* 415–1085 mg/kg, New Zealand *L. scoparium* 403–1296 mg/kg, *L. polygalifolium* 652–2003 mg/kg, *L. whitei* 465–1074 mg/kg, *L. speciosum* 279–442 mg/kg, *L. liversidgei* 263–1603 mg/kg) indicating all had a Non-Peroxide Activity (NPA) of 10+ [13] (Table 1).

### Comparison of manuka vs non-manuka honey aromatic profiles by HPLC-DAD

The aromatic profiles of manuka honey were considerably more complex than non-manuka honey, reflecting a high UV absorbance and diversity of compounds. The peak intensity (mAU) of aromatic compounds in grey ironbark honey was approximately 12-fold lower than Australian *L. scoparium* manuka honey (Fig 1). Due to this lower content, identifying aromatic compounds by HPLC-DAD in non-*Leptospermum* honeys requires alternative extraction techniques, as previously shown by Moore, Brooks [56].

**Fig 1:**
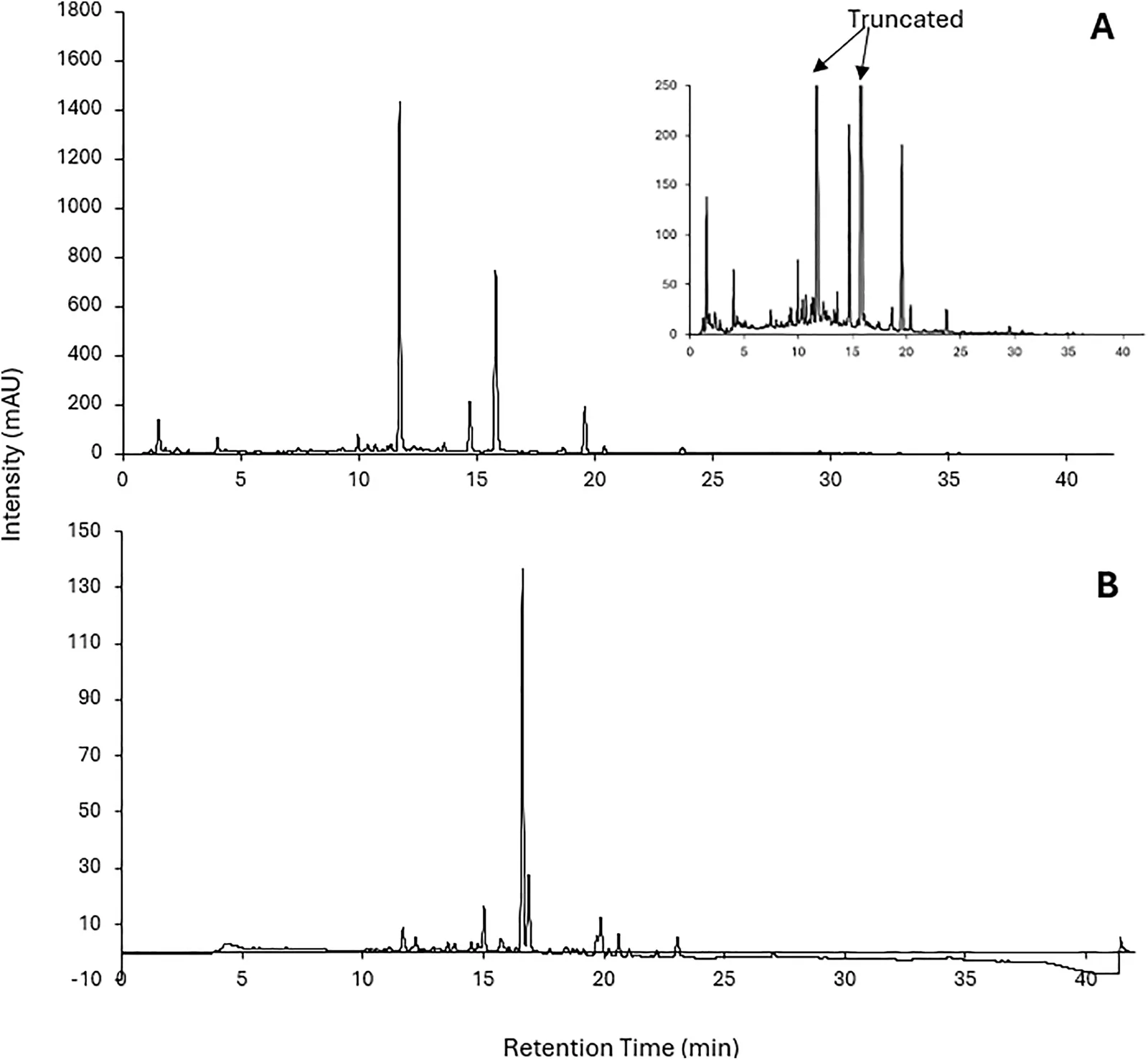
Comparison of peak intensity in manuka vs non-manuka honey analysed by HPLC-DAD at 220 nm. (**A**) Manuka (Australian *L. scoparium)* with zoom insert. (**B**) Grey Ironbark (*E. paniculata)*.

### HPLC-DAD comparison of concentrated and non-concentrated (diluted) honey extracts

Organic solvent extraction fails to fully recover polar compounds, such as kojic acid (a non-aromatic compound) and leptosperin, owing to their preferential solubility in the aqueous phase [59,60]. While the qualitative aromatic profile remains consistent between diluted (20% m/v) and concentrated extraction methods, quantitative differences are observed (Fig 2). Diluted extraction methods were employed for HPLC-DAD analysis of aromatic profiles, while concentrated extractions were used to prepare samples for the 15-LOX assay, which required the removal of sugars. Fig 2 presents HPLC chromatograms of *L. scoparium* honey (LS7) prepared using both extraction methods. For direct comparison, the concentrated extract was subsequently diluted to an equivalent concentration of 20% (m/v) whole honey to match the conditions of the diluted extraction protocol. The concentrated extraction trace showed considerably fewer sugars than the diluted extract. Despite partial loss of aromatic compounds during concentrated extraction, the overall aromatic profiles remain consistent for 15-LOX testing.

**Fig 2:**
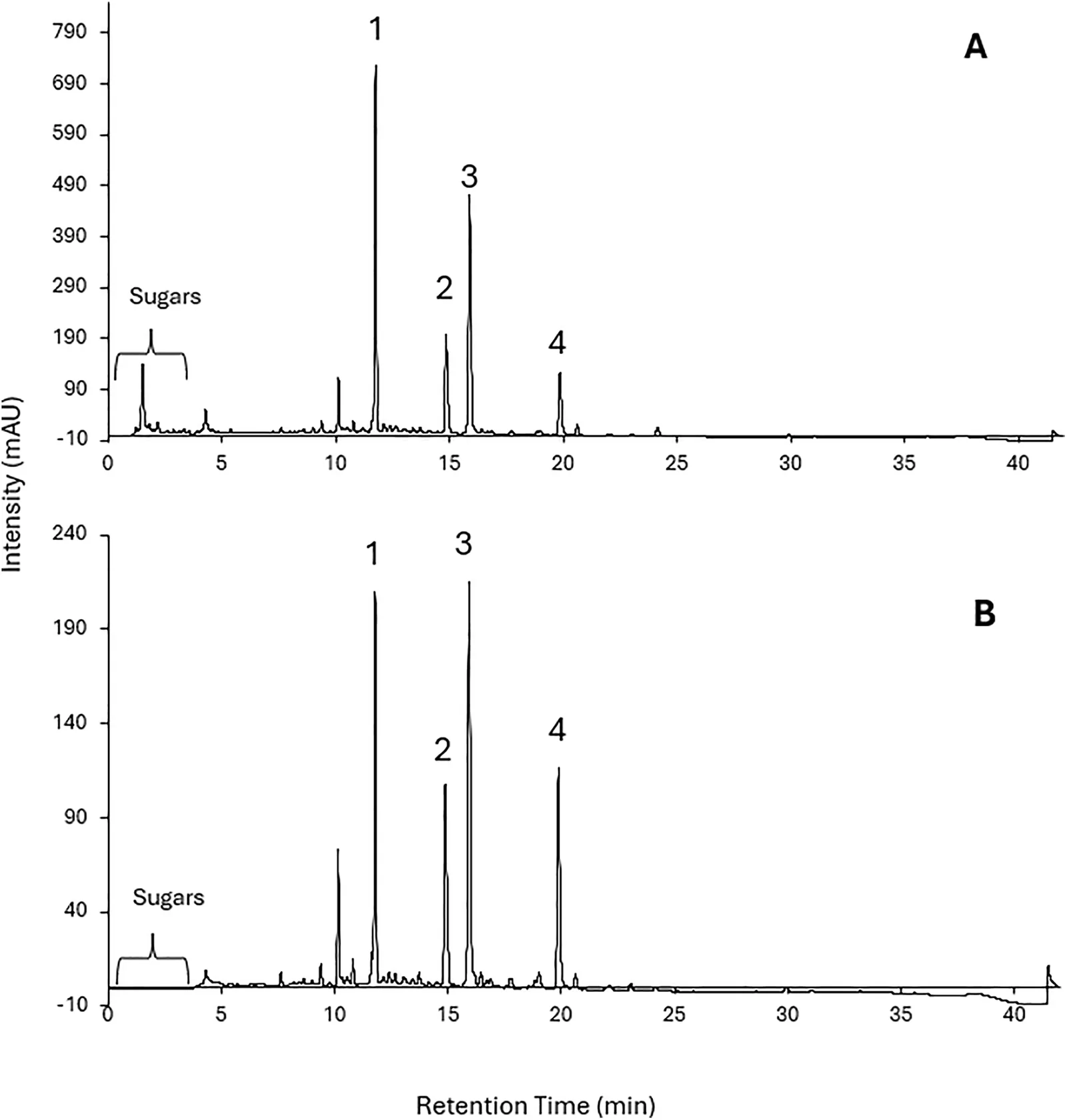
HPLC-DAD chromatograms of *L. scoparium* honey illustrating aromatic compound recovery using different extraction methods at 220 nm. (**A**) Diluted (20% m/v). (**B**) Concentrated aromatic extraction diluted to equivalent 20% m/v. Labelled peaks 1: Leptosperin, 2: 3-Phenyllactic acid, 3: 2-Methoxybenzoic acid and 4: Methyl Syringate.

### Aromatic constituents of manuka honeys

The first aim of this study was to identify the aromatic profile of different *Leptospermum* (manuka) honey varieties and compare the total aromatic content using HPLC-DAD analysis. As part of the broader effort to define Australian manuka honey, this paper identified marker compounds that were consistent across the major Australian manuka honey varietals. Notably, this paper is the first to report the aromatic content of *L. liversidgei*. *L. speciosum* and *L. whitei* honeys. To achieve this, an in-house aromatic library comprised of 110 target compounds was used to identify the compounds present in manuka honey samples (S1 Table).

Of the 110 compounds screened, 27 were detected in manuka honey varieties (S2 Table). Among these, nine compounds were consistently present across all samples analysed: Kojic Acid (KojA) (non-aromatic), DL-*p*-hydroxyphenyllactic acid (DL-*p*-HPLA), epigallocatechin (EGC), lepteridine (Leptd) (heteroaromatic), leptosperin (Lepp), 3-phenyllactic acid (3-PLA), 4-methoxyphenyllactic acid (4-MPLLA), 2-methoxybenzoic acid (2-MBA) and methyl syringate (MS). While kojic acid, *p*-hydroxybenzoic acid, and methyl syringate are commonly found in non-manuka honeys, the combined presence of DL-*p*-hydroxyphenyllactic acid, lepteridine, leptosperin, 3-phenyllactic acid, and 2-methoxybenzoic acid is considered characteristic of manuka honeys [34,61,62]. The present study extends these findings by demonstrating, that *L*. *whitei*, *L. speciosum* and *L. liversidgei* honeys, also share a core chemical fingerprint despite quantitative differences between species. Fig 3 shows the HPLC traces of the six Australian manuka varietals and one New Zealand manuka honey. Supplementary Table 2 & Supplementary Data 7 presents the concentrations (mg/kg) of individual compounds identified by HPLC-DAD in manuka samples, along with the total aromatic content, FC-TPC and 15-LOX IC_50_ values for each sample.

**Fig 3:**
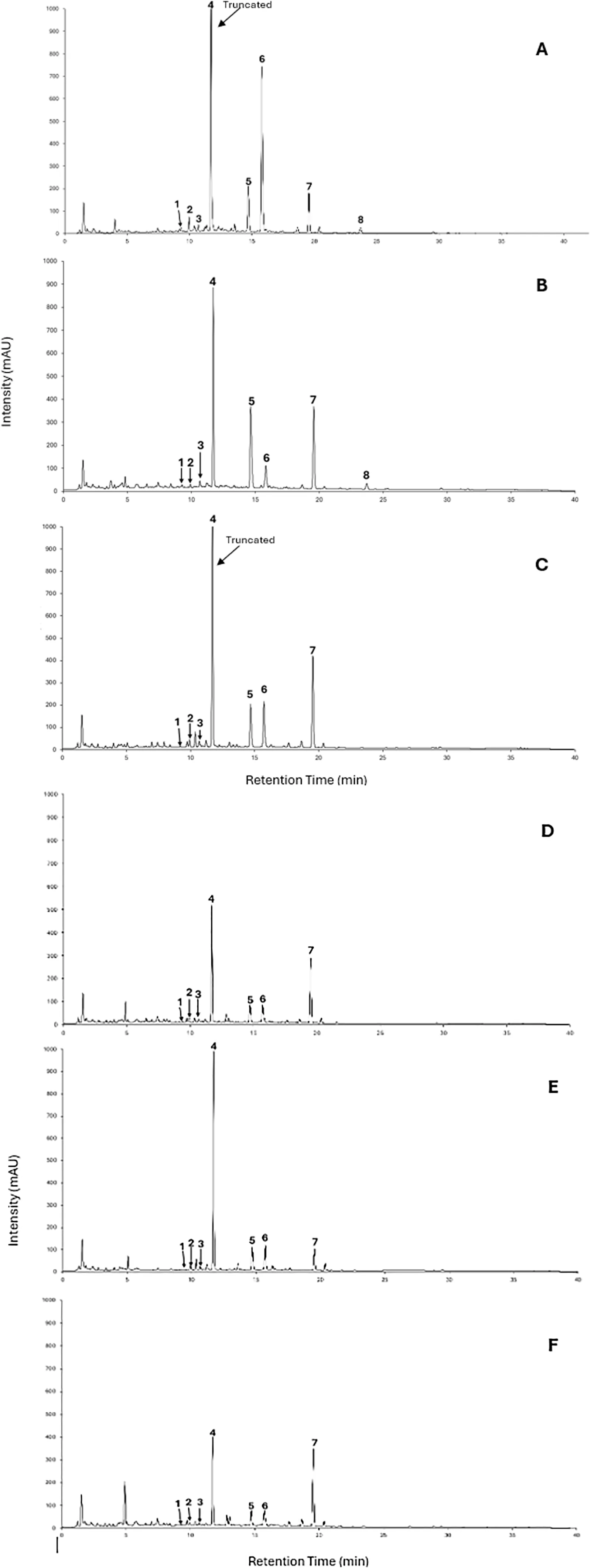
Aromatic profile of monofloral manuka honey varietals analysed by HPLC-DAD at 220 nm. Marker compounds- 1: DL-*p*-hydroxyphenyllactic acid, 2: Epigallocatechin, 3: Lepteridine, 4: Leptosperin, 5: 3-Phenyllactic acid, 6: 2- Methoxybenzoic acid, 7: Methyl syringate and 8: 2- Methoxyacetophenone. HPLC trace of different manuka honeys (A) *L. scoparium* (Aust), (B) *L. scoparium* (NZ), (C) *L. polygalifolium* (Aust), (D) *L. whitei* (Aust), (E) *L. speciosum* (Aust) and (F) *L. liversidgei* (Aust).

### Concentrations of aromatic compounds in *Leptospermum* honey varieties

Post hoc comparisons using Tukey’s HSD test (S1 Dataset) showed leptosperin concentrations differed significantly among manuka honey varieties (S2 Table). *L. liversidgei* honey (290.9 ± 63.6 mg/kg) contained significantly lower leptosperin levels than *L. polygalifolium* (1086.9 ± 212.9 mg/kg; p = 0.005) and Australian *L. scoparium* honeys (936.0 ± 120.0 mg/kg; p = 0.023). *L. polygalifolium* contained significantly higher leptosperin concentrations compared with *L. whitei* (481.0 ± 68.2 mg/kg; p = 0.047) and *L. speciosum* (379.3 ± 245.9 mg/kg; p = 0.047). No significant differences were detected between Australian (936.0 ± 120.0 mg/kg) and New Zealand *L. scoparium* (671.0 ± 83.6 mg/kg) honeys with regards to leptosperin, nor were there significant differences among the remaining honey types.

Leptosperin is a characteristic marker of manuka honey and is generally abundant in *L. scoparium* derived honeys. The leptosperin concentrations measured in the present study were consistent with previous findings by Bong, Prijic [63], who observed levels of 918 ± 54 mg/kg and 734 ± 62 mg/kg in Tasmanian and New Zealand *L. scoparium* honey, respectively. In addition, the elevated leptosperin levels found in the six *L. polygalifolium* honeys (1086.9 ± 212.9 mg/kg) were broadly comparable to previously reported findings (1578 ± 264 mg/kg) [63], indicating that this species of manuka may represent an equally rich source of this bioactive compound. Conversely, the relatively low concentrations in *L. liversidgei* suggest species-specific differences in nectar chemistry.

2-Methoxybenzoic acid was detected in all manuka honey samples studied, with significant variation among floral sources (S2 Dataset; S2 Table). *L. liversidgei* (84.2 ± 14.1 mg/kg) contained significantly lower 2-MBA concentrations compared to *L. polygalifolium* (186.9 ± 41.3 mg/kg; p = 0.022) but did not differ significantly from other groups (*p* > 0.05). *L. polygalifolium* contained significantly higher 2-MBA concentrations than *L. whitei* (89.1 ± 14.8 mg/kg; p = 0.032), *L. speciosum* (63.8 ± 22.0 mg/kg; p = 0.018), and *L. scoparium* honeys from both Australia (14.0 ± 4.2 mg/kg; p < 0.001) and New Zealand (13.1 ± 2.4 mg/kg; p < 0.001). This finding is consistent with a previous study by Beitlich, Koelling-Speer [30], who also reported notably higher levels of 2-MBA in *L. polygalifolium* than *L. scoparium*, suggesting that elevated 2-MBA may be a characteristic feature of *L. polygalifolium* relative to other manuka varieties. No significant differences were detected between Australian and New Zealand *L. scoparium* honeys.

A statistically significant difference in 3-phenyllactic acid concentrations was observed between New Zealand *L. scoparium* honey (1039.2 ± 70.1 mg/kg) and all Australian manuka honey varieties (p < 0.001) (S2 Table; S3 Dataset). In contrast, no significant differences in 3-PLA content were detected among the Australian manuka honeys. This is broadly consistent with Bong, Loomes [64] who also quantified 3-phenyllactic acid in New Zealand *L. scoparium* honey (740 ± 43 mg/kg) using HPLC-DAD, supporting its presence as a major constituent of *L. scoparium* honey. Although, the higher concentration observed in the present study suggests variability related to floral source quality and seasonal conditions of the honeys analysed.

Methyl syringate concentrations also varied significantly among honey types (S2 Table; S4 Dataset). *L. polygalifolium* honey (146.9 ± 22.6 mg/kg) contained significantly higher levels than *L. liversidgei* (79.0 ± 19.7 mg/kg; p = 0.028), *L. whitei* (83.3 ± 8.0 mg/kg; p = 0.045), *L. speciosum* (21.0 ± 1.0 mg/kg; p < 0.001), and Australian *L. scoparium* (81.3 ± 9.4 mg/kg; p = 0.019) honeys. Methyl syringate has previously been reported at high concentrations in *L. polygalifolium* honey (233.82 mg/kg) [32]. In addition, New Zealand *L. scoparium* (126.7 ± 6.3 mg/kg) honey contained significantly higher methyl syringate concentrations than *L. speciosum* (21.0 ± 1.0 mg/kg) honey (p = 0.002). No significant difference was observed between Australian and New Zealand *L. scoparium* honeys, nor any other pairwise comparisons.

### Total aromatic content by HPLC-DAD analysis

Total aromatic content among manuka honey varieties was compared using HPLC-DAD analysis. Fig 4 illustrates the total aromatic compound concentrations across the different honey species. Australian and New Zealand *L. scoparium* honeys contained comparable amounts of total aromatic compounds (2184.8 ± 201.8 mg/kg and 2197.7 ± 134.8 mg/kg, respectively). Both Australian and New Zealand *L. scoparium* honeys contained significantly greater total aromatic compound levels than *L. liversidgei* (772.4 ± 91.6 mg/kg; p < 0.001), *L. whitei* (1135.3 ± 198.5 mg/kg; p = 0.014 and 0.017, respectively), and *L. speciosum* (716.6 ± 290.1 mg/kg; p = 0.003 for both) (S5 Dataset). In addition, *L. polygalifolium* honey (1926.1 ± 298.0 mg/kg) contained higher total aromatic content than *L. liversidgei* (p = 0.008) and *L. speciosum* (p = 0.021) honeys.

**Fig 4:**
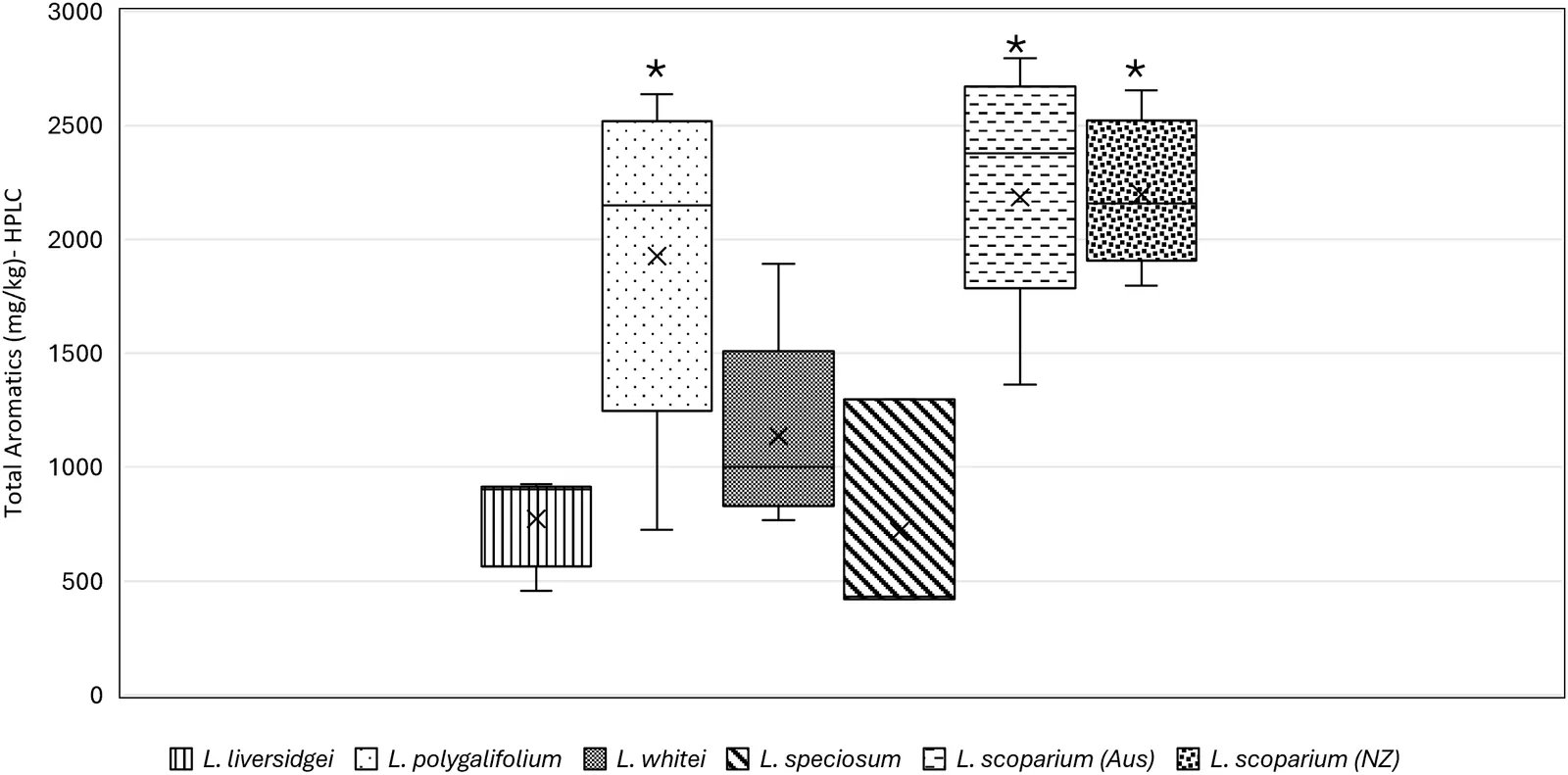
Box plot showing the concentration of total aromatic compounds of different manuka honeys from Aust (n = 26) and NZ (n = 6), determined using HPLC-DAD analysis. *L. polygalifolium*, *L. scoparium* (Aust), and *L. scoparium* (NZ) are significantly different from *L. liversidgei*, *L. whitei*, and *L. speciosum* (p < 0.05), as indicated by *.

### Total phenolic content by Folin-Ciocalteu assay

To compare the total phenolic content of different manuka honeys, the Folin-Ciocalteu assay was utilised (Fig 5). New Zealand *L. scoparium* honey (345.8 ± 18.8 mg/kg GAE) contained a greater amount of total phenolic compounds than Australian *L. scoparium* (205.1 ± 14.4 mg/kg GAE; p = 0.002), *L. whitei* (200.5 ± 18.1 mg/kg GAE; p = 0.004), and *L. speciosum* (146.7 mg/kg GAE; p < 0.001) honey*. L. polygalifolium* honey (287.3 ± 38.5 mg/kg GAE) contained a greater amount of total phenolic compounds than *L. speciosum* honey (p = 0.021) but was not significantly different from New Zealand *L. scoparium* honey (S5 Dataset). The amount of total phenolic compounds in *L. liversidgei* honey (257.2 ±27.1 mg/kg GAE) was not significantly different to any of the other manuka honey. *L. speciosum* honey consistently ranked lowest amongst manuka honey for amount of total aromatic and total phenolic compounds, determined using HPLC-DAD and FC-TPC methods respectively. This apparent disparity is likely attributable to fundamental differences between the two methods.

**Fig 5:**
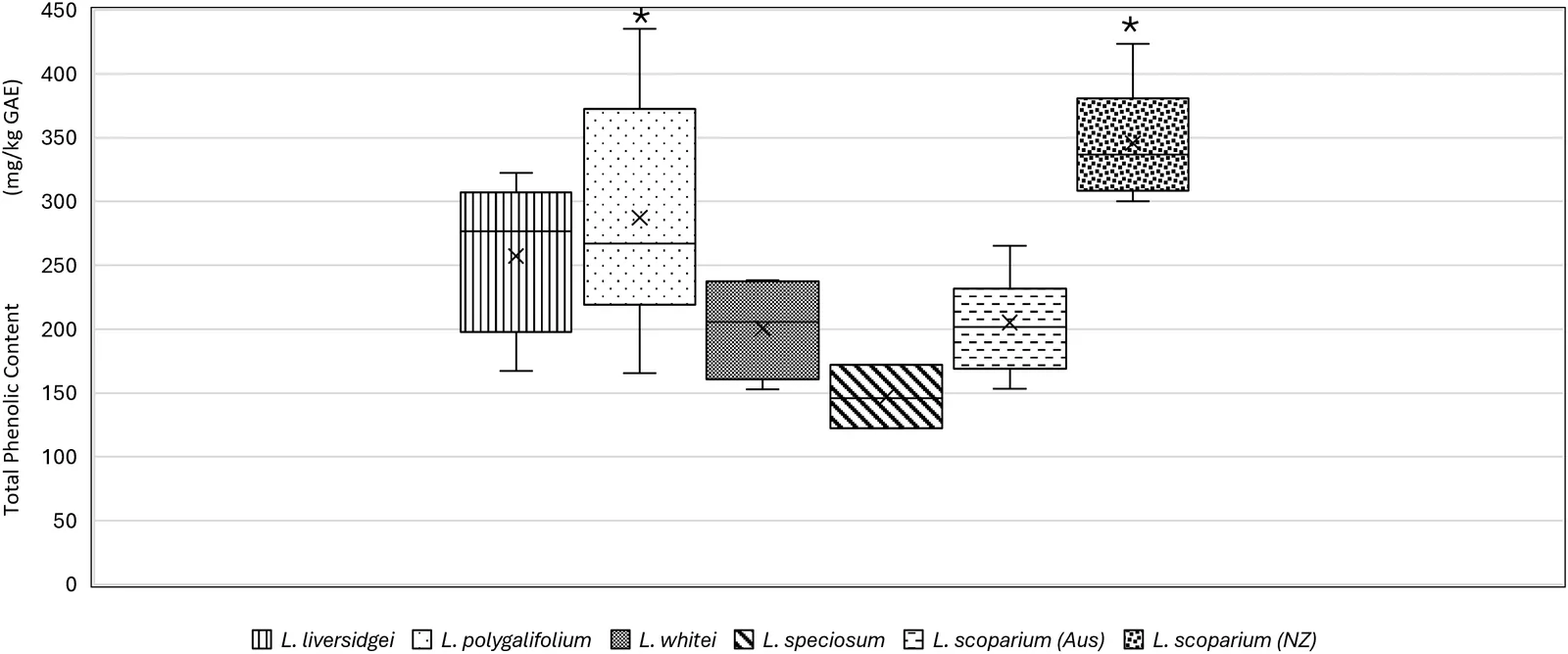
Box plot illustrating the concentration of total phenolic compounds of different manuka honeys from Aust (n = 26) and NZ (n = 6), determined using the FC-TPC assay. *L. polygalifolium* and *L. scoparium* (NZ) are significantly different from *L. scoparium* (Aust), *L. liversidgei*, *L. whitei*, and *L. speciosum* (p < 0.05), as indicated by *.

Manuka honeys from Australia and New Zealand contain high levels of aromatic compounds, including leptosperin, lepteridine, 3-phenyllactic acid, 4-methoxyphenyllactic acid, 2-methoxyacetophenone, 4-hydroxyphenyllactic acid, methyl syringate and syringic acid. However, only the latter three compounds are classified as phenolics, in that they contain a hydroxyl group attached to an aromatic ring. The FC-TPC method selectively reflects total phenolic content based on reducing capacity and does not account for aromatic compounds lacking phenolic functional groups, whereas HPLC–DAD enables the detection and quantification of a broader range of aromatic constituents, including non-phenolic aromatic compounds [65,66]. Consequently, honeys enriched in non-phenolic aromatic compounds may rank higher when assessed by HPLC–DAD but lower in FC-TPC based analyses.

The FC total phenolic content measurements were compared to HPLC-DAD measurements of aromatics (phenolic + non-phenolic aromatic compounds) (Fig 6). As expected, FC-TPC results did not correlate with total aromatics, exhibiting an R^2^ of 0.116 (Fig 6). Previous studies have frequently used FC-TPC as a surrogate measure of antioxidant capacity in manuka honey. Total phenolic content is expressed as gallic acid equivalents (GAE mg/kg); however, this approach also assumes that all phenolic constituents exhibit similar reactivity to gallic acid. Gallic acid possesses three hydroxyl groups, giving it a strong reducing capacity and therefore a greater response in the FC-TPC assay [67,68]. In contrast, many phenolic compounds that are present in manuka honey such as methyl syringate, contain fewer free hydroxyl groups and may have lower reactivity toward the reagent. As a result, these compounds may contribute disproportionately less to the measured FC-TPC value [67,68], despite their potential biological relevance. Consequently, the FC-TPC method, which reports phenolic content relative to gallic acid, is likely to underestimate the concentration of aromatic compounds within the honey. The HPLC-DAD method of analysis does not use gallic acid as a comparator, potentially providing a more reliable measure of total aromatic content than the FC-TPC method used for total phenolic content.

**Fig 6:**
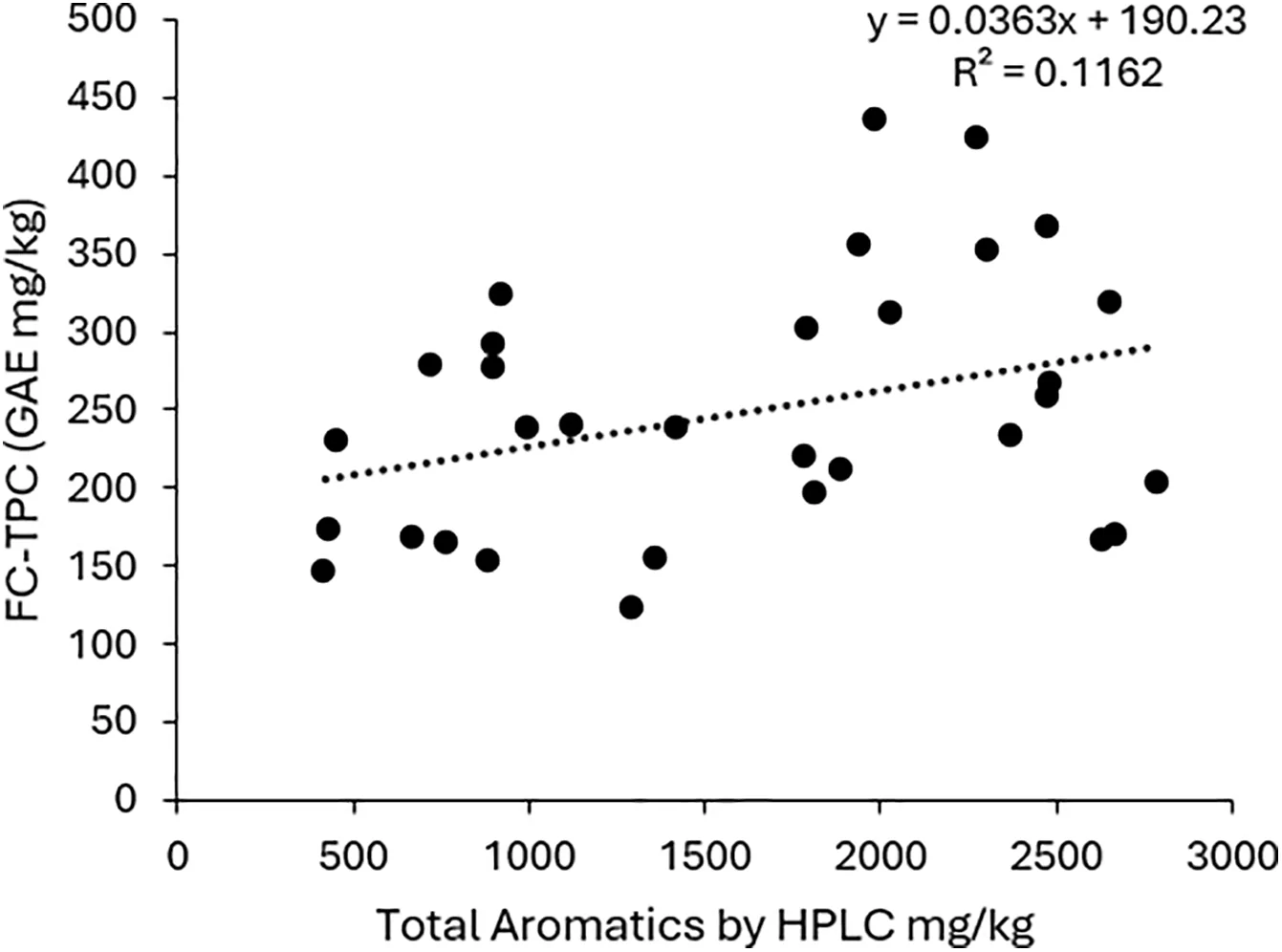
Comparison of FC total phenolic content measurements with HPLC-DAD quantified aromatic compounds.

### 15- LOX assay

The final aim of this study was to investigate the potency of sugar-free aromatic extracts of *Leptospermum* honeys to inhibit soybean 15-lipoxygenase (15-LOX) activity using a cell-free assay. Lipoxygenases are non-haem iron-containing enzymes that catalyse the oxidation of polyunsaturated fatty acids to produce lipid hydroperoxides, which serve as precursors to numerous pro-inflammatory lipid mediators, including leukotrienes [69]. Consequently, inhibition of LOX enzymes has been investigated as a therapeutic strategy to reduce oxidative stress and inflammation, and many naturally occurring plant-derived compounds have been reported to inhibit LOX activity [69,70]. 15-LOX is one of several isoforms within the lipoxygenase enzyme family [71]. Eugenol, p-chlorophenol and other related phenolic aromatic compounds have been shown to inhibit lipoxygenase activity by suppressing the formation of 15-HETE (15-Hydroxyeicosatetraenoic acid), a product of 15-LOX metabolism [72]. To our knowledge, this is the first study to investigate the effects of manuka honey on 15-LOX inhibition.

The potency of aromatic compounds from honey extracts to inhibit 15-LOX activity is reported in Fig 7 where 100% represents the starting stock solutions (extract of 20.0 g of honey/1.0 mL of 50% aqueous ethanol), which were then run as serial dilutions. This data identified that both Australian *L. scoparium* (*IC*_*50*_ = 13.2%) *and* New Zealand *L. scoparium* (*IC*_*50*_ = 13.1%) were more potent than *L. liversidgei* (*IC*_*50*_ = 37.5%) (p < 0.001) and *L. speciosum* (*IC*_*50*_ = 36.6%) (p = 0.007) (S6 Dataset). No other differences were observed between *Leptospermum* honey varieties. In contrast to manuka honeys, which reduced 15-LOX activity to ~7–24% of controls in the absence of honey extract, non-manuka honeys were without effect at concentrations up to the 100% stock solution (Fig 7; S6 Dataset). Interestingly, total phenolic content did not correlate with enzyme inhibition, whereas linear regression analysis showed a moderate negative correlation between aromatic content and 15-LOX IC_50_values (R^2^ = 0.4042) (Fig 8). This suggests that samples with higher aromatic compound concentrations generally exhibited lower IC_50_ values, reflecting stronger 15-LOX inhibitory activity However, no correlation was observed between individual aromatic compounds or total phenolic content (TPC) and IC_50_ values.

**Fig 7:**
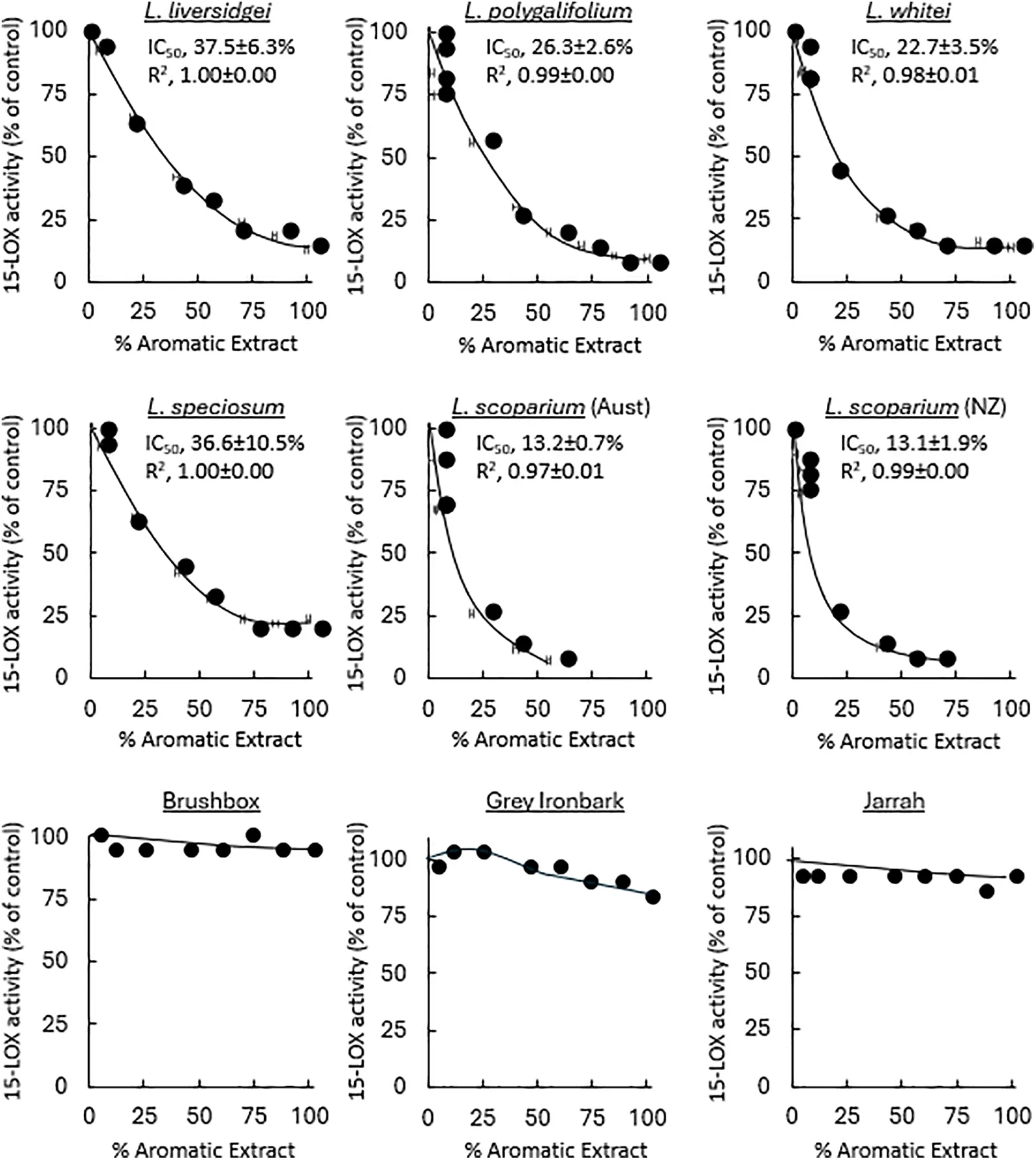
Mean (±SEM) inhibitory effects of manuka and non-manuka aromatic extracts on soybean 15-LOX activity in a cell free assay.

**Fig 8:**
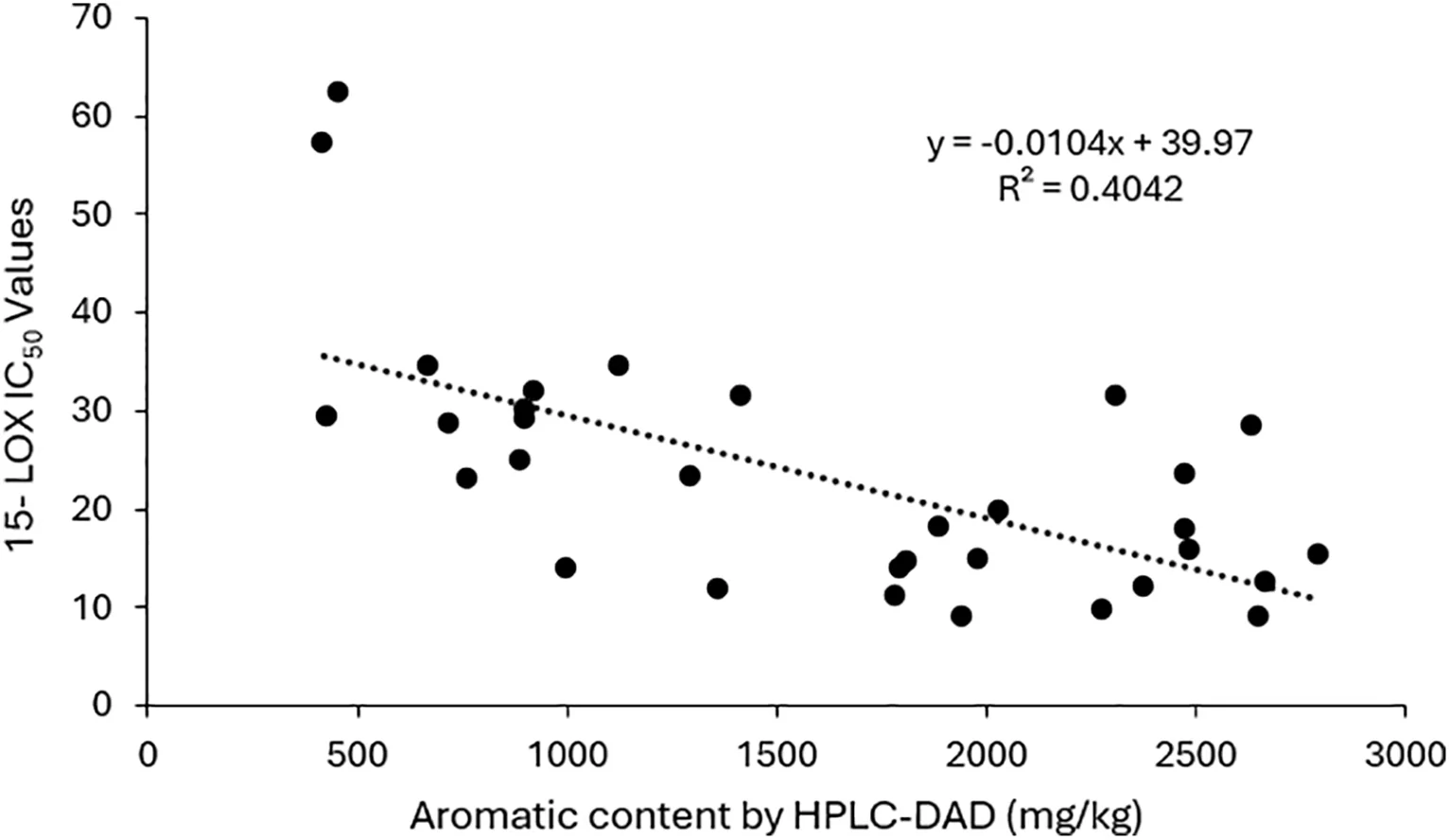
Correlation between 15-LOX IC₅₀ values and total aromatic content determined by HPLC-DAD (mg/kg) in Manuka honey samples.

## Conclusion

This study demonstrates that Manuka honeys are rich in aromatic compounds, with both *L. scoparium* (from Australia and New Zealand) and *L. polygalifolium* varieties exhibiting the highest total aromatic and phenolic contents. HPLC-DAD analysis provides a complete profile of a honey’s aromatic composition, accounting for both phenolic and non-phenolic compounds, compared to the Folin-Ciocalteu assay. Sugar-free aromatic extracts of manuka honey effectively inhibited 15-LOX, with both Australian and New Zealand *L. scoparium* extracts showing the greatest activity and anti-inflammatory potentials. Correlation analysis further indicated that total aromatic content was moderately associated with 15-LOX inhibition. Overall, these findings highlight the importance of non-phenolic aromatic compounds in contributing to the bioactivity of manuka honey and suggest that HPLC-DAD-based profiling offers a more accurate reflection of honey’s potential health benefits than conventional phenolic assays alone. Further investigation is needed to determine whether manuka extracts will provide therapeutic benefits for medical conditions in which oxidative stress and inflammation are implicated.

## Supporting information

S1 Table List of 110 chemical standards with CAS registry numbers and supplier details. (DOCX)

S2 Table Quantification of aromatic compounds found in Australian & New Zealand manuka honey types, with corresponding Folin-Ciocalteu-Total Phenolic Content and 15-LOX IC_50_ values. (DOCX)

S1 Dataset Leptosperin concentrations vs. *Leptospermum* species. (DOCX)

S2 Dataset 2- MBA concentrations vs. *Leptospermum* species. (DOCX)

S3 Dataset 3-PLA concentrations vs. *Leptospermum* species. (DOCX)

S4 Dataset MS concentrations vs. *Leptospermum* species. (DOCX)

S5 Dataset Statistical analyses of HPLC and FC-TPC total aromatic content of different *Leptospermum* species. (XLSX)

S6 Dataset LOX IC_50_ raw data and calculation. (XLSX)

S7 Manuka Aromatics raw data spreadsheet. (XLSX)

## Abbreviations

ACN: Acetonitrile
EtOH: Ethanol
TFA: Trifluoroacetic acid
MilliQ: Ultrapure water
MPA: Mobile phase A
MPB: Mobile phase B
DAD: Diode array detection
HPLC: High-performance liquid chromatography
KojA: Kojic acid
GA: Gallic acid
Pter: Pteridine
SyrA: Syringic acid
EGC: Epigallocatechin
MandA: Mandelic acid
Leptd: Lepteridine
p-HBA: p-Hydroxybenzoic acid
4-HPAA: 4-Hydroxyphenylacetic acid
Lepp: Leptosperin
SPE: Solid phase extraction
FC-TPC: Folin-Ciocalteu total phenolic content assay
3-PLA: 3-Phenyllactic acid
4-MPLLA: 4-Methoxyphenyllactic acid
2-MBA: 2-Methoxybenzoic acid
BA: Benzoic acid
Lum: Lumichrome
PAA: Phenylacetic acid
3,4,5-TMBA: 3,4,5-Trimethoxybenzoic acid
4-MPLA: 4-Methoxyphenylacetic acid
Geni: Genistin
MS: Methyl syringate
Lute: Luteolin
2-MAP: 2’-Methoxyacetophenone
Pinob: Pinobanksin
Chr: Chrysin
Pinoc: Pinocembrin
4-MMA: 4-Methoxymandelic acid
DL-p-HPLA: DL-p-Hydroxyphenyllactic acid
Aust: Australia/Australian
15-LOX: 15-Lipoxygenase
