# Supplementary material for: Aromatic profiles and 15-LOX inhibitory effects of Australian and New Zealand Manuka honeys
Source: PLoS One. 2026 Nov 3;21(11):e0361877. doi: 10.1371/journal.pone.0361877 (PMC13730423; doi:10.1371/journal.pone.0361877)
Supplement: Supplementary file 1 [file pone.0361877.s001.docx]

Supplementary Table 1: List of 110 chemical standards with CAS registry numbers and supplier details.

| **Chemical Standard** | **CAS #** | **Supplier** |
| --- | --- | --- |
| Catechin hydrate | 154-23-4 | Sigma-Aldrich |
| Epicatechin | 490-46-0 | Sigma-Aldrich |
| (-)- Epigallocatechin gallate | 989-51-5 | Collaborator (University of Western Australia) |
| Epigallocatechin | 95 989-51-5 | Collaborator (University of Western Australia) |
| Hesperidin | 520-26-3 | Sigma-Aldrich |
| Eriodictyol | 4049-38-1 | Sigma-Aldrich |
| Naringenin | 480-41-1 | Sigma-Aldrich |
| Hesperetin | 69097-99-0 | Sigma-Aldrich |
| Pinocembrin | 480-39-7 | Sigma-Aldrich |
| Sakuranetin | 2957-21-3 | Collaborator (University of Western Australia) |
| 6-Hydroxyflavone-beta-D-glucoside | 128401-92-3 | Sigma-Aldrich |
| Luteolin | 491-70-3 | Sigma-Aldrich |
| Apigenin | 520-36-5 | Sigma-Aldrich |
| 5-Methoxyflavone | 42079-78-7 | Alfa Aesar |
| Chrysin | 480-40-0 | Sigma-Aldrich |
| Acacetin | 480-44-4 | Sigma-Aldrich |
| Genkwanin (4’,5-Dihydroxy-7-methoxyflavone) | 437-64-9 | Alfa Aesar |
| 3-*o*-methylquercitin | 1486-70-0 | Sigma-Aldrich |
| Tectochrysin | 520-28-5 | Sigma-Aldrich |
| Vitexin | 3681-93-4 | Sigma-Aldrich |
| 6,2',4'-Trimethoxyflavone | 720675-90-1 | Sigma-Aldrich |
| Tricetin | 520-31-0 | MolPort |
| Rutin hydrate | 207671-50-9 | Sigma-Aldrich |
| Myricetin | 529-44-2 | Sigma-Aldrich |
| Fisetin | 528-48-3 | Sigma-Aldrich |
| Quercetin | 117-39-5 | Sigma-Aldrich |

Supplementary Table 1: List of 110 chemical standards with CAS registry numbers and supplier details (continued).

| **Chemical Standard** | **CAS #** | **Supplier** |
| --- | --- | --- |
| Kaempferol | 520-18-3 | Sigma-Aldrich |
| Isorhamnetin (Quercetin 3'-methyl ether) | 480-19-3 | Sigma-Aldrich |
| Galangin | 548-83-4 | Sigma-Aldrich |
| Taxifolin | 480-18-2 | Collaborator (University of Western Australia) |
| *p*-Anisaldehyde | 123-11-5 | Sigma-Aldrich |
| 3,4-Dihydroxybenzaldehyde | 102-32-9 | Sigma-Aldrich |
| Vanillin | 121-33-5 | Sigma-Aldrich |
| 2-Methoxybenzoic acid | 579-75-9 | Sigma-Aldrich |
| Methyl *p*-Hydroxybenzoate (Methylparaben) | 99-76-3 | Sigma-Aldrich |
| Benzoic acid | 65-85-0 | Sigma-Aldrich |
| 3,4,5-Trimethoxybenzoic acid (Eudesmic acid) | 118-41-2 | Sigma-Aldrich |
| *o*-Toluic acid | 118-90-1 | Sigma-Aldrich |
| *p*-Toluic acid | 99-94-5 | Sigma-Aldrich |
| *m*-Toluic acid | 99-04-7 | Sigma-Aldrich |
| 2-Methoxyacetophenone | 4079-52-1 | Sigma-Aldrich |
| 2-Hydroxyacetophenone | 582-24-1 | Sigma-Aldrich |
| Methyl 3,4,5-trimethoxybenzoate | 1916-07-0 | Sigma-Aldrich |
| Salicylic acid | 69-72-7 | Sigma-Aldrich |
| Pinobanksin | 548-82-3 | Collaborator (University of Western Australia) |
| 2,3,4-Trihydroxybenzoic acid | 97 610-02-6 | Collaborator (University of Western Australia) |
| Pyrogallol | 87-66-1 | Fluka |
| Gallic acid | 149-91-7 | Sigma-Aldrich |
| 3,4-Dihydroxybenzoic acid | 99-50-3 | Sigma-Aldrich |
| 3,5-Dihydroxybenzoic acid | 99-10-5 | Sigma-Aldrich |
| Pyrocatechol | 120-80-9 | Collaborator (University of Western Australia) |
| *p*-Hydroxybenzoic acid | 99-96-7 | Sigma-Aldrich |

Supplementary Table 1: List of 110 chemical standards with CAS registry numbers and supplier details (continued).

| **Chemical Standard** | **CAS #** | **Supplier** |
| --- | --- | --- |
| Ellagic acid | 476-66-4 | Collaborator (University of Western Australia) |
| Syringic acid | 530-57-4 | Sigma-Aldrich |
| Vanillic acid | 121-34-6 | Sigma-Aldrich |
| m-Hydroxybenzoic acid | 99-06-9 | Sigma-Aldrich |
| 2,5-Dihydroxybenzoic acid (Gentisic acid) | 490-79-9 | Sigma-Aldrich |
| 2,4,5-Trimethoxybenzoic acid | 490-64-2 | Sigma-Aldrich |
| 2,3,4-Trimethoxybenzoic acid | 573-11-5 | Sigma-Aldrich |
| Methyl syringate | 884-35-5 | Sigma-Aldrich |
| Methyl vanillate | 3943-74-6 | Sigma-Aldrich |
| Resorcylic acid | 303-38-8 | Sigma-Aldrich |
| Cryptochlorogenic acid | 905-99-7 | Sigma-Aldrich |
| Chlorogenic acid | 327-97-9 | Sigma-Aldrich |
| Caffeic acid (3,4-Dihydroxycinnamic acid) | 331-39-5 | Sigma-Aldrich |
| Isoferulic acid | 537-73-5 | Sigma-Aldrich |
| *m*-Coumaric acid | 14755-02-3 | Sigma-Aldrich |
| *o*-Coumaric acid | 614-60-8 | Sigma-Aldrich |
| Caffeic acid dimethyl ether | 2316-26-9 | Sigma-Aldrich |
| Rosmarinic acid | 20283-92-5 | Sigma-Aldrich |
| Trans-cinnamic acid | 140-10-3 | Sigma-Aldrich |
| Methyl trans *p*-coumarate | 19367-38-5 | Sigma-Aldrich |
| 4-Methoxycinnamic acid | 943-89-5 | Collaborator (University of Western Australia) |
| Methyl ferulate | 22329-76-6 | Sigma-Aldrich |
| Caffeic acid phenethyl ester | 104594-70-9 | Sigma-Aldrich |
| Artepillin C | 72944-19-5 | Sigma-Aldrich |
| Neochlorogenic acid | 906-33-2 | Sigma-Aldrich |
| *p*-Coumaric acid | 501-98-4 | Sigma-Aldrich |

Supplementary Table 1: List of 110 chemical standards with CAS registry numbers and supplier details (continued).

| **Chemical Standard** | **CAS #** | **Supplier** |
| --- | --- | --- |
| Sinapic acid | 530-59-6 | Sigma-Aldrich |
| Ferulic acid (4-Hydroxy-3-Methoxycinnamic acid) | 537-98-4 | Sigma-Aldrich |
| 4-Methoxyphenylacetic acid | 104-01-8 | Collaborator (University of Western Australia) |
| Homogentisic acid | 415-13-8 | Sigma-Aldrich |
| 3,4-Dihydroxyphenylacetic acid | 102-32-9 | Sigma-Aldrich |
| DL-*p*-Hydroxyphenyllactic acid | 306-23-0 | Sigma-Aldrich |
| 4-Hydroxyphenylacetic acid | 156-38-7 | Sigma-Aldrich |
| Homovanillic acid | 306-08-1 | Sigma-Aldrich |
| 4-Hydroxyphenylpropanoic acid (Phloretic acid) | 501-97-3 | Collaborator (University of Western Australia) |
| Mandelic acid | 90-64-2 | Collaborator (University of Western Australia) |
| Phenylacetic acid | 103-82-2 | Fluka |
| 3-Phenyllactic acid | 828-01-3 | Sigma-Aldrich |
| 4-Methoxyphenyllactic acid | 28030-15-1 | Collaborator (University of Western Australia) |
| Daidzein | 486-66-8 | Sigma-Aldrich |
| Genistein | 446-72-0 | Sigma-Aldrich |
| Formononetin | 485-72-3 | Sigma-Aldrich |
| Biochanin A (5,7-Dihydroxy-4’-methoxyisoflavone) | 491-80-5 | Sigma-Aldrich |
| Genistin | 529-59-9 | Sigma-Aldrich |
| *p*-Methoxyphenol | 150-76-5 | Fluka |
| Hydroxymethylfurfural | 67-47-0 | Sigma-Aldrich |
| Pteridine | 91-18-9 | Sigma-Aldrich |
| Lepteridine | N/A | University of Auckland |
| 2-Furylmethylketone (2-Acetylfuran) | 1192-62-7 | Sigma-Aldrich |
| Lumichrome | 1086-80-2 | Sigma-Aldrich |
| Abscisic acid | 21293-29-8 | Sigma-Aldrich |
| Kojic acid | 501-30-4 | Sigma-Aldrich |

Supplementary Table 1: List of 110 chemical standards with CAS registry numbers and supplier details (continued).

| **Chemical Standard** | **CAS #** | **Supplier** |
| --- | --- | --- |
| Isopsuedocumenol (2,3,5-Trimethylphenol) | 697-82-5 | Sigma-Aldrich |
| Acetophenone | 98-86-2 | Sigma-Aldrich |
| Thymol | 89-83-8 | Sigma-Aldrich |
| Leptosperin | N/A | Collaborator (Isolated from Australian *Leptospermum* honey) |
| 4-Methylpyrocatechol | 452-86-8 | Sigma-Aldrich |
| 4-Methoxymandelic acid (2-hydroxy-2-(4-methoxyphenyl)acetic acid) | 10502-44-0 | Sigma-Aldrich |
