# Supplementary material for: Aromatic profiles and 15-LOX inhibitory effects of Australian and New Zealand Manuka honeys
Source: PLoS One. 2026 Nov 3;21(11):e0361877. doi: 10.1371/journal.pone.0361877 (PMC13730423; doi:10.1371/journal.pone.0361877)
Supplement: Supplementary file 2 [file pone.0361877.s002.docx]

S2 Table: Quantification of aromatic compounds found in Australian & New Zealand manuka honey types, with corresponding Folin-Ciocalteu-Total Phenolic Content and 15-LOX IC_50_ values. ‘ND’ indicates compounds not detected.

| Sample ID | *Leptospermum scoparium* (Aust) | | | | | | | |
| --- | --- | --- | --- | --- | --- | --- | --- | --- |
|  | **LS1** | **LS2** | **LS3** | **LS4** | **LS5** | | **LS6** | **LS7** |
| KojA | 18.3 | 43.5 | 30.4 | 39.7 | 27.5 | 27.8 | | 36.2 |
| GA | ND | 0.3 | 0.4 | ND | ND | 0.5 | | 0.6 |
| Pter | ND | ND | ND | ND | ND | ND | | ND |
| DL-*p*-HPLA | 5.5 | 8.8 | 14.0 | 8.9 | 12.4 | 13.4 | | 9.7 |
| EGC | 48.4 | 19.0 | 36.0 | 30.5 | 24.2 | 24.9 | | 56.0 |
| MandA | 3.9 | 9.1 | 15.8 | ND | ND | 20.9 | | 9.8 |
| Leptd | 7.0 | 9.4 | 16.6 | 13.6 | 17.4 | 18.3 | | 9.5 |
| *p*-HBA | 2.1 | 1.7 | 2.6 | 1.5 | 1.5 | 12.3 | | 2.2 |
| 4-HPAA | 10.2 | 13.6 | 24.8 | 13.1 | 18.4 | 25.8 | | 19.1 |
| Lepp | 1240.0 | 523.0 | 1143.0 | 729.2 | 1063.5 | 1273.0 | | 580.0 |
| 4-MMA | ND | 36.4 | 63.0 | ND | ND | 69.7 | | 29.2 |
| SyrA | 2.5 | ND | ND | 2.3 | 4.4 | ND | | 1.7 |
| 3-PLA | 722.0 | 204.7 | 427.8 | 198.2 | 293.6 | 334.7 | | 505.4 |
| 4-MPLLA | 241.7 | 410.7 | 786.4 | 627.3 | 764.9 | 841.0 | | 468.5 |
| 2-MBA | 39.2 | 7.6 | 11.1 | 8.3 | 10.4 | 12.0 | | 9.1 |
| BA | ND | ND | ND | ND | ND | ND | | ND |
| Lum | 4.9 | 0.7 | ND | 9.6 | ND | ND | | 1.6 |
| PAA | 19.5 | ND | ND | ND | ND | ND | | 2.0 |
| 3,4,5-TMBA | 3.1 | 1.4 | 2.1 | ND | ND | 2.0 | | 2.6 |
| 4-MPLA | ND | ND | 1.3 | ND | ND | ND | | ND |
| Geni | 1.7 | 1.6 | 2.7 | 3.0 | 2.8 | 3.4 | | 3.1 |
| MS | 106.2 | 52.8 | 68.3 | 87.5 | 116.2 | 86.3 | | 51.9 |
| Lute | 0.9 | 0.9 | 3.0 | ND | 1.2 | 2.2 | | 1.4 |
| 2-MAP | 9.4 | 9.8 | 18.6 | 11.2 | 17.2 | 22.5 | | 13.9 |
| Pinob | 1.7 | 3.0 | 0.9 | 0.9 | 1.1 | 1.9 | | 0.6 |
| Chr | ND | 1.1 | 0.3 | ND | ND | 0.6 | | ND |
| Pinoc | 0.9 | 2.4 | 1.3 | 1.0 | 0.8 | 1.3 | | 0.7 |
| Aromatic Totals (mg/kg) | **2489.1** | **1361.5** | **2670.4** | **1785.8** | **2377.5** | **2794.5** | | **1814.8** |
| FC-TPC (GAE mg/kg) | **265.5** | **153.4** | **168.9** | **219.7** | **231.8** | **201.6** | | **195.1** |
| 15- LOX IC_50_ values (% of stock solutions) | **15.6** | **11.7** | **12.5** | **11.1** | **11.9** | **15.2** | | **14.4** |

Supplementary Table 2 (continued): Quantification of aromatic compounds found in Australian & New Zealand manuka honey types, with corresponding Folin-Ciocalteu Total Phenolic Content and 15-LOX IC_50_ values. ‘ND’ indicates compounds not detected.

| Sample ID | *Leptospermum scoparium* (New Zealand) | | | | | |
| --- | --- | --- | --- | --- | --- | --- |
|  | **NZ1** | **NZ2** | **NZ3** | **NZ4** | **NZ5** | **NZ6** |
| KojA | 17.4 | 15.3 | 54.3 | 66.3 | 66.1 | 11.2 |
| GA | ND | ND | ND | ND | ND | ND |
| Pte | 4.5 | 10.4 | 8.4 | 11.8 | 5.9 | 7.9 |
| DL-*p*-HPLA | 5.7 | 8.5 | 9.9 | 9.4 | 8.4 | 10.4 |
| EGC | 6.3 | 10.6 | 9.5 | 10.2 | 8.7 | 10.4 |
| MandA | ND | ND | ND | ND | ND | ND |
| Leptd | 9 | 16.4 | 24.4 | 15 | 15.6 | 13.9 |
| *p*-HBA | 2.6 | ND | ND | 0.1 | ND | ND |
| 4-HPAA | ND | ND | ND | ND | ND | ND |
| Lepp | 366 | 588.2 | 898 | 543.6 | 816.3 | 816.3 |
| 4-MMA | ND | ND | ND | ND | ND | ND |
| SyrA | 1.3 | ND | 2.1 | 1.7 | 1.8 | 2.5 |
| 3-PLA | 794.5 | 914 | 1112.1 | 1057.1 | 1061.9 | 1295.8 |
| 4-MPLLA | 435.1 | 318 | 157 | 46.9 | 104.9 | 314.8 |
| 2-MBA | 7.5 | 11.2 | 12.5 | 14.4 | 23.8 | 9.1 |
| BA | ND | ND | ND | ND | ND | ND |
| Lum | 2.0 | 1.6 | 1.1 | 1.4 | 1.4 | 0.5 |
| PAA | 2.0 | 2.3 | 2.3 | 2.0 | 1.6 | 2.0 |
| 3,4,5-TMBA | 0.2 | 0.2 | 0.4 | 0.5 | 0.6 | ND |
| 4-MPLA | ND | ND | ND | ND | ND | ND |
| Geni | 3.4 | 1.0 | 1.3 | 0.7 | 2.0 | 3.4 |
| MS | 117.2 | 101.2 | 145 | 128.3 | 134.3 | 134 |
| Lute | ND | ND | ND | ND | ND | ND |
| 2-MAP | 11.2 | 21.4 | 24.5 | 25.4 | 17.4 | 11.2 |
| Pinob | 4.7 | 6.5 | 9.8 | 3.4 | 3.3 | 4.7 |
| Chr | 1.5 | 2.4 | 0.6 | 1.5 | 1.4 | 2.1 |
| Pinoc | 4.4 | 5.8 | 4.6 | 4.4 | 2.9 | 4.3 |
| Aromatic Totals (mg/kg) | **1796.5** | **2035.0** | **2477.8** | **1944.1** | **2278.3** | **2654.5** |
| FC-TPC (GAE mg/kg) | **300.2** | **311.2** | **366.5** | **355** | **423.4** | **318.3** |
| 15- LOX IC_50_ values (% of stock solutions) | **13.7** | **19.6** | **17.8** | **8.8** | **9.7** | **9.0** |

Supplementary Table 2 (continued): Quantification of aromatic compounds found in Australian & New Zealand manuka honey types, with corresponding Folin-Ciocalteu Total Phenolic Content and 15-LOX IC_50_ values. ‘ND’ indicates compounds not detected.

| Sample ID | *Leptospermum polygalifolium* (Aust) | | | | | |
| --- | --- | --- | --- | --- | --- | --- |
|  | **LP1** | **LP2** | **LP3** | **LP4** | **LP5** | **LP6** |
| KojA | 17.1 | 10.0 | 5.1 | 12.8 | 10.4 | 4.1 |
| GA | 3.2 | ND | ND | ND | ND | 9.8 |
| Pter | ND | 2.0 | 2.3 | 4.5 | 2.2 | 4.2 |
| DL-*p*-HPLA | 1.8 | 0.5 | 1.2 | 1.3 | 0.6 | 1.4 |
| EGC | 47.1 | 9.2 | 8.0 | 23.7 | 14.1 | 16.4 |
| MandA | 10.1 | 21.0 | 10.3 | 29.7 | 16.2 | ND |
| Leptd | 15.3 | 16.6 | 5.3 | 14.1 | 9.4 | 14.6 |
| *p*-HBA | 1.2 | 1.7 | 2.9 | 1.1 | 2.0 | 1.2 |
| 4-HPAA | ND | ND | ND | ND | ND | ND |
| Lepp | 1702.0 | 1455.0 | 300.0 | 1390.0 | 767.0 | 907.3 |
| 4-MMA | ND | ND | ND | ND | ND | ND |
| SyrA | 2.0 | 1.5 | 9.1 | 5.4 | 6.3 | 3.2 |
| 3-PLA | 134.7 | 649.3 | 137.8 | 515.3 | 293.7 | 470.0 |
| 4-MPLLA | 19.2 | 9.7 | 23.7 | 23.1 | 10.7 | 10.6 |
| 2-MBA | 123.1 | 319.2 | 38.8 | 236.0 | 153.4 | 250.8 |
| BA | 3.0 | 7.8 | ND | 7.5 | 2.5 | 6.4 |
| Lum | 5.9 | 0.6 | 0.7 | 0.5 | 0.3 | ND |
| PAA | 2.2 | 9.8 | 46.4 | 54.8 | 22.8 | 47.0 |
| 3,4,5-TMBA | 1.6 | ND | 0.4 | 0.6 | ND | 0.2 |
| 4-MPLA | 0.4 | 0.4 | 27.7 | 29.8 | 12.2 | 25.9 |
| Geni | 0.3 | 0.1 | 1.7 | 0.7 | 0.6 | ND |
| MS | 220.9 | 122.3 | 102.3 | 127.3 | 95.8 | 212.5 |
| Lute | 0.7 | ND | ND | ND | 0.4 | ND |
| 2-MAP | ND | ND | ND | ND | ND | ND |
| Pinob | ND | ND | ND | ND | ND | ND |
| Chr | ND | ND | ND | ND | ND | ND |
| Pinoc | ND | ND | ND | ND | ND | ND |
| Aromatic Totals (mg/kg) | **2311.8** | **2636.7** | **723.7** | **2478.2** | **1420.6** | **1985.6** |
| FC-TPC (GAE mg/kg) | **351.5** | **165.4** | **277.5** | **256.9** | **236.8** | **435.4** |
| 15- LOX IC_50_ values (% of stock solutions) | **31.4** | **28.4** | **28.5** | **23.5** | **31.4** | **14.8** |

Supplementary Table 2 (continued): Quantification of aromatic compounds found in Australian & New Zealand manuka honey types, with corresponding Folin-Ciocalteu Total Phenolic Content and 15-LOX IC_50_ values. ‘ND’ indicates compounds not detected.

| Sample ID | *Leptospermum whitei* (Aust) | | | | |
| --- | --- | --- | --- | --- | --- |
|  | **LW1** | **LW2** | **LW3** | **LW4** | **LW5** |
| KojA | 10.7 | 9.0 | 8.1 | 6.5 | 9.3 |
| GA | 0.6 | 1.2 | ND | 1.5 | ND |
| Pter | 1.6 | 2.7 | 1.3 | 1.8 | 1.4 |
| DL-*p*-HPLA | 2.5 | 0.8 | 0.6 | 1.0 | 1.8 |
| EGC | 23.4 | 9.6 | 14.2 | 9.1 | 132.1 |
| MandA | 21.2 | 12.3 | 13.9 | ND | 74.9 |
| Leptd | 6.5 | 10.7 | 4.6 | 7.1 | 9.2 |
| *p*-HBA | 3.2 | 1.5 | 2.5 | 1.8 | 1.4 |
| 4-HPAA | ND | ND | ND | ND | ND |
| Lepp | 261.0 | 471.0 | 428.0 | 650.6 | 594.5 |
| 4-MMA | ND | ND | ND | ND | ND |
| SyrA | 1.9 | 1.1 | 4.3 | 4.2 | 2.0 |
| 3-PLA | 268.8 | 216.7 | 218.1 | 192.2 | 817.4 |
| 4-MPLLA | 27.3 | 34.6 | 8.4 | 8.1 | 3.0 |
| 2-MBA | 46.4 | 93.1 | 73.1 | 96.6 | 136.4 |
| BA | 2.6 | ND | 2.1 | 2.0 | 3.4 |
| Lum | 0.9 | 1.1 | 1.0 | ND | 3.8 |
| PAA | 18.2 | 21.6 | 16.1 | 27.2 | 15.0 |
| 3,4,5-TMBA | 0.5 | ND | ND | ND | 2.3 |
| 4-MPLA | 12.4 | 12.6 | 9.3 | 15.8 | 7.5 |
| Geni | 1.0 | 0.9 | 1.8 | ND | ND |
| MS | 56.7 | 99.7 | 82.1 | 100.0 | 77.9 |
| Lute | 0.4 | ND | ND | ND | ND |
| 2-MAP | ND | ND | ND | ND | ND |
| Pinob | ND | ND | ND | ND | ND |
| Chr | ND | ND | ND | ND | ND |
| Pinoc | ND | ND | ND | ND | ND |
| Aromatic Totals (mg/kg) | **767.8** | **1000.2** | **889.5** | **1125.5** | **1893.3** |
| FC -TPC (GAE mg/kg) | **163.4** | **237.3** | **152.8** | **238.3** | **210.7** |
| 15- LOX IC_50_ values (% of stock solutions) | **23** | **13.7** | **24.7** | **34.3** | **18** |

Supplementary Table 2 (continued): Quantification of aromatic compounds found in Australian & New Zealand manuka honey types, with corresponding Folin-Ciocalteu Total Phenolic Content and 15-LOX IC_50_ values. ‘ND’ indicates compounds not detected.

| Sample ID | *Leptospermum speciosum* (Aust) | | |
| --- | --- | --- | --- |
|  | **LSp1** | **LSp2** | **LSp3** |
| KojA | 7.8 | 8.0 | 5.2 |
| GA | ND | ND | ND |
| Pter | ND | ND | ND |
| DL-*p*-HPLA | 1.3 | 0.9 | 0.3 |
| EGC | 27.9 | 22.2 | 11.2 |
| MandA | 19.8 | 18.6 | 9.3 |
| Leptd | 3.4 | 3.0 | 8.1 |
| *p*-HBA | 1.6 | 3.5 | 2.3 |
| 4-HPAA | 4.8 | 4.2 | ND |
| Lepp | 120.0 | 147.0 | 870.8 |
| 4-MMA | ND | ND | ND |
| SyrA | ND | ND | ND |
| 3-PLA | 165.6 | 126.0 | 248.3 |
| 4-MPLLA | 11.9 | 9.4 | 4.8 |
| 2-MBA | 39.5 | 44.1 | 107.8 |
| BA | ND | ND | ND |
| Lum | 3.1 | 4.2 | 3.4 |
| PAA | 4.0 | 6.2 | 5.3 |
| 3,4,5-TMBA | ND | ND | 0.3 |
| 4-MPLA | ND | ND | ND |
| Geni | 0.5 | 0.6 | 0.3 |
| MS | 20.7 | 22.9 | 19.3 |
| Lute | 0.3 | ND | ND |
| 2-MAP | ND | ND | ND |
| Pinob | ND | ND | ND |
| Chr | ND | ND | ND |
| Pinoc | ND | ND | ND |
| Aromatic Totals (mg/kg) | **432.2** | **420.8** | **1296.7** |
| FC-TPC (GAE mg/kg) | **172** | **145.8** | **122.2** |
| 15- LOX IC_50_ values (% of stock solutions) | **29.2** | **57.2** | **23.2** |

Supplementary Table 2 (continued): Quantification of aromatic compounds found in Australian & New Zealand manuka honey types, with corresponding Folin-Ciocalteu Total Phenolic Content and 15-LOX IC_50_ values. ‘ND’ indicates compounds not detected.

| Sample ID | *Leptospermum liversidgei* (Aust) | | | | |
| --- | --- | --- | --- | --- | --- |
|  | **LL1** | **LL2** | **LL3** | **LL4** | **LL5** |
| KojA | 12.6 | 12.7 | 3.8 | 3.8 | 5.9 |
| GA | 0.8 | ND | ND | 2.3 | ND |
| Pter | ND | 4.0 | 2.8 | 2.7 | 3.5 |
| DL-*p*-HPLA | 1.9 | 1.1 | 1.2 | 0.9 | ND |
| EGC | 3.9 | 3.9 | 7.0 | 6.8 | 8.6 |
| MandA | 22.8 | ND | ND | ND | ND |
| Leptd | 5.6 | 17.9 | 6.3 | 6.3 | 17.4 |
| *p*-HBA | 2.2 | 1.7 | 1.7 | 1.7 | 2.4 |
| 4-HPAA | 3.0 | ND | ND | ND | ND |
| Lepp | 71.0 | 240.0 | 414.8 | 408.6 | 319.9 |
| 4-MMA | ND | ND | ND | ND | ND |
| SyrA | ND | ND | 7.3 | 9.2 | 4.8 |
| 3-PLA | 200.2 | 155.1 | 173.5 | 181.9 | 223.0 |
| 4-MPLLA | 35.7 | 107.3 | 13.0 | 21.0 | 72.7 |
| 2-MBA | 58.0 | 71.3 | 75.4 | 77.3 | 139.0 |
| BA | ND | ND | ND | 1.4 | ND |
| Lum | 2.9 | 6.7 | 0.8 | 1.5 | 3.4 |
| PAA | 2.2 | 6.3 | 38.9 | 49.6 | 9.4 |
| 3,4,5-TMBA | ND | ND | 0.6 | 0.7 | 0.3 |
| 4-MPLA | 1.5 | 7.4 | 32.5 | 31.4 | 7.2 |
| Geni | 0.5 | 1.3 | ND | ND | 1.3 |
| MS | 32.1 | 34.9 | 123.6 | 118.6 | 85.6 |
| Lute | ND | ND | ND | ND | ND |
| 2-MAP | ND | ND | ND | ND | ND |
| Pinob | ND | ND | ND | ND | ND |
| Chr | ND | ND | ND | ND | ND |
| Pinoc | ND | ND | ND | ND | ND |
| Aromatic Totals (mg/kg) | **456.9** | **671.6** | **903.2** | **925.7** | **904.4** |
| FC Total Phenolic content (GAE mg/kg) | **228.3** | **167.4** | **291.6** | **322.3** | **276.5** |
| 15- LOX IC_50_ values (% of stock solutions) | **62.3** | **34.3** | **29.1** | **31.9** | **29.9** |
