# Supplementary material for: Aromatic profiles and 15-LOX inhibitory effects of Australian and New Zealand Manuka honeys
Source: PLoS One. 2026 Nov 3;21(11):e0361877. doi: 10.1371/journal.pone.0361877 (PMC13730423; doi:10.1371/journal.pone.0361877)
Supplement: Supplementary file 3 [file pone.0361877.s003.docx]

Leptosperin concentrations vs *Leptospermum* species

**Oneway**

[DataSet0]

| **ANOVA** | | | | | |
| --- | --- | --- | --- | --- | --- |
| VAR00002 | | | | | |
|  | Sum of Squares | df | Mean Square | F | Sig. |
| Between Groups | 2675220.157 | 5 | 535044.031 | 5.131 | .002 |
| Within Groups | 2711264.052 | 26 | 104279.387 |  |  |
| Total | 5386484.209 | 31 |  |  |  |

| **ANOVA Effect Sizes**^a,b^ | | | | |
| --- | --- | --- | --- | --- |
|  | | Point Estimate | 95% Confidence Interval | |
|  |  |  | Lower | Upper |
| VAR00002 | Eta-squared | .497 | .106 | .612 |
|  | Epsilon-squared | .400 | -.065 | .538 |
|  | Omega-squared Fixed-effect | .392 | -.063 | .530 |
|  | Omega-squared Random-effect | .114 | -.012 | .184 |
| a. Eta-squared and Epsilon-squared are estimated based on the fixed-effect model. | | | | |
| b. Negative but less biased estimates are retained, not rounded to zero. | | | | |

**Post Hoc Tests**

1= L. Liversidgei

2= L. polygala

3= L. whitei

4= L. speciosum

5= L. scoparium AUST

6= L. scoparium NZ

| **Multiple Comparisons** | | | | | | |
| --- | --- | --- | --- | --- | --- | --- |
| Dependent Variable: VAR00002 | | | | | | |
| Tukey HSD | | | | | | |
| (I) VAR00001 | (J) VAR00001 | Mean Difference (I-J) | Std. Error | Sig. | 95% Confidence Interval | |
|  |  |  |  |  | Lower Bound | Upper Bound |
| 1.00 | 2.00 | -796.02333^*^ | 195.53970 | .005 | -1396.8103 | -195.2364 |
|  | 3.00 | -190.16000 | 204.23456 | .935 | -817.6615 | 437.3415 |
|  | 4.00 | -88.40667 | 235.82975 | .999 | -812.9830 | 636.1696 |
|  | 5.00 | -645.09714^*^ | 189.08446 | .023 | -1226.0506 | -64.1436 |
|  | 6.00 | -380.54000 | 195.53970 | .399 | -981.3269 | 220.2469 |
| 2.00 | 1.00 | 796.02333^*^ | 195.53970 | .005 | 195.2364 | 1396.8103 |
|  | 3.00 | 605.86333^*^ | 195.53970 | .047 | 5.0764 | 1206.6503 |
|  | 4.00 | 707.61667^*^ | 228.34118 | .047 | 6.0487 | 1409.1847 |
|  | 5.00 | 150.92619 | 179.65788 | .957 | -401.0646 | 702.9169 |
|  | 6.00 | 415.48333 | 186.43979 | .259 | -157.3445 | 988.3112 |
| 3.00 | 1.00 | 190.16000 | 204.23456 | .935 | -437.3415 | 817.6615 |
|  | 2.00 | -605.86333^*^ | 195.53970 | .047 | -1206.6503 | -5.0764 |
|  | 4.00 | 101.75333 | 235.82975 | .998 | -622.8230 | 826.3296 |
|  | 5.00 | -454.93714 | 189.08446 | .191 | -1035.8906 | 126.0164 |
|  | 6.00 | -190.38000 | 195.53970 | .922 | -791.1669 | 410.4069 |
| 4.00 | 1.00 | 88.40667 | 235.82975 | .999 | -636.1696 | 812.9830 |
|  | 2.00 | -707.61667^*^ | 228.34118 | .047 | -1409.1847 | -6.0487 |
|  | 3.00 | -101.75333 | 235.82975 | .998 | -826.3296 | 622.8230 |
|  | 5.00 | -556.69048 | 222.83817 | .161 | -1241.3507 | 127.9698 |
|  | 6.00 | -292.13333 | 228.34118 | .793 | -993.7013 | 409.4347 |
| 5.00 | 1.00 | 645.09714^*^ | 189.08446 | .023 | 64.1436 | 1226.0506 |
|  | 2.00 | -150.92619 | 179.65788 | .957 | -702.9169 | 401.0646 |
|  | 3.00 | 454.93714 | 189.08446 | .191 | -126.0164 | 1035.8906 |
|  | 4.00 | 556.69048 | 222.83817 | .161 | -127.9698 | 1241.3507 |
|  | 6.00 | 264.55714 | 179.65788 | .684 | -287.4336 | 816.5479 |
| 6.00 | 1.00 | 380.54000 | 195.53970 | .399 | -220.2469 | 981.3269 |
|  | 2.00 | -415.48333 | 186.43979 | .259 | -988.3112 | 157.3445 |
|  | 3.00 | 190.38000 | 195.53970 | .922 | -410.4069 | 791.1669 |
|  | 4.00 | 292.13333 | 228.34118 | .793 | -409.4347 | 993.7013 |
|  | 5.00 | -264.55714 | 179.65788 | .684 | -816.5479 | 287.4336 |
| *. The mean difference is significant at the 0.05 level. | | | | | | |

**Homogeneous Subsets**

| **VAR00002** | | | | |
| --- | --- | --- | --- | --- |
| Tukey HSD^a,b^ | | | | |
| VAR00001 | N | Subset for alpha = 0.05 | | |
|  |  | 1 | 2 | 3 |
| 1.00 | 5 | 290.8600 |  |  |
| 4.00 | 3 | 379.2667 | 379.2667 |  |
| 3.00 | 5 | 481.0200 | 481.0200 | 481.0200 |
| 6.00 | 6 | 671.4000 | 671.4000 | 671.4000 |
| 5.00 | 7 |  | 935.9571 | 935.9571 |
| 2.00 | 6 |  |  | 1086.8833 |
| Sig. |  | .450 | .106 | .065 |
| Means for groups in homogeneous subsets are displayed. | | | | |
| a. Uses Harmonic Mean Sample Size = 4.961. | | | | |
| b. The group sizes are unequal. The harmonic mean of the group sizes is used. Type I error levels are not guaranteed. | | | | |
