# Supplementary material for: Aromatic profiles and 15-LOX inhibitory effects of Australian and New Zealand Manuka honeys
Source: PLoS One. 2026 Nov 3;21(11):e0361877. doi: 10.1371/journal.pone.0361877 (PMC13730423; doi:10.1371/journal.pone.0361877)
Supplement: Supplementary file 4 [file pone.0361877.s004.docx]

2- MBA concentrations vs *Leptospermum* species

**Explore**

[DataSet0]

**VAR00001**

| **Case Processing Summary** | | | | | | | |
| --- | --- | --- | --- | --- | --- | --- | --- |
|  | VAR00001 | Cases | | | | | |
|  |  | Valid | | Missing | | Total | |
|  |  | N | Percent | N | Percent | N | Percent |
| VAR00002 | 1.00 | 5 | 100.0% | 0 | 0.0% | 5 | 100.0% |
|  | 2.00 | 6 | 100.0% | 0 | 0.0% | 6 | 100.0% |
|  | 3.00 | 5 | 100.0% | 0 | 0.0% | 5 | 100.0% |
|  | 4.00 | 3 | 100.0% | 0 | 0.0% | 3 | 100.0% |
|  | 5.00 | 7 | 100.0% | 0 | 0.0% | 7 | 100.0% |
|  | 6.00 | 6 | 100.0% | 0 | 0.0% | 6 | 100.0% |

| **Descriptives** | | | | | |
| --- | --- | --- | --- | --- | --- |
|  | VAR00001 | | | Statistic | Std. Error |
| VAR00002 | 1.00 | Mean | | 84.2000 | 14.10840 |
|  |  | 95% Confidence Interval for Mean | Lower Bound | 45.0288 |  |
|  |  |  | Upper Bound | 123.3712 |  |
|  |  | 5% Trimmed Mean | | 82.6111 |  |
|  |  | Median | | 75.4000 |  |
|  |  | Variance | | 995.235 |  |
|  |  | Std. Deviation | | 31.54735 |  |
|  |  | Minimum | | 58.00 |  |
|  |  | Maximum | | 139.00 |  |
|  |  | Range | | 81.00 |  |
|  |  | Interquartile Range | | 43.50 |  |
|  |  | Skewness | | 1.903 | .913 |
|  |  | Kurtosis | | 4.021 | 2.000 |
|  | 2.00 | Mean | | 186.8833 | 41.28099 |
|  |  | 95% Confidence Interval for Mean | Lower Bound | 80.7672 |  |
|  |  |  | Upper Bound | 292.9995 |  |
|  |  | 5% Trimmed Mean | | 187.7593 |  |
|  |  | Median | | 194.7000 |  |
|  |  | Variance | | 10224.722 |  |
|  |  | Std. Deviation | | 101.11737 |  |
|  |  | Minimum | | 38.80 |  |
|  |  | Maximum | | 319.20 |  |
|  |  | Range | | 280.40 |  |
|  |  | Interquartile Range | | 165.88 |  |
|  |  | Skewness | | -.246 | .845 |
|  |  | Kurtosis | | -.708 | 1.741 |
|  | 3.00 | Mean | | 89.1200 | 14.81355 |
|  |  | 95% Confidence Interval for Mean | Lower Bound | 47.9910 |  |
|  |  |  | Upper Bound | 130.2490 |  |
|  |  | 5% Trimmed Mean | | 88.8667 |  |
|  |  | Median | | 93.1000 |  |
|  |  | Variance | | 1097.207 |  |
|  |  | Std. Deviation | | 33.12412 |  |
|  |  | Minimum | | 46.40 |  |
|  |  | Maximum | | 136.40 |  |
|  |  | Range | | 90.00 |  |
|  |  | Interquartile Range | | 56.75 |  |
|  |  | Skewness | | .276 | .913 |
|  |  | Kurtosis | | .719 | 2.000 |
|  | 4.00 | Mean | | 63.8000 | 22.04004 |
|  |  | 95% Confidence Interval for Mean | Lower Bound | -31.0306 |  |
|  |  |  | Upper Bound | 158.6306 |  |
|  |  | 5% Trimmed Mean | | . |  |
|  |  | Median | | 44.1000 |  |
|  |  | Variance | | 1457.290 |  |
|  |  | Std. Deviation | | 38.17447 |  |
|  |  | Minimum | | 39.50 |  |
|  |  | Maximum | | 107.80 |  |
|  |  | Range | | 68.30 |  |
|  |  | Interquartile Range | | . |  |
|  |  | Skewness | | 1.704 | 1.225 |
|  |  | Kurtosis | | . | . |
|  | 5.00 | Mean | | 13.9571 | 4.24785 |
|  |  | 95% Confidence Interval for Mean | Lower Bound | 3.5630 |  |
|  |  |  | Upper Bound | 24.3513 |  |
|  |  | 5% Trimmed Mean | | 12.9079 |  |
|  |  | Median | | 10.4000 |  |
|  |  | Variance | | 126.310 |  |
|  |  | Std. Deviation | | 11.23875 |  |
|  |  | Minimum | | 7.60 |  |
|  |  | Maximum | | 39.20 |  |
|  |  | Range | | 31.60 |  |
|  |  | Interquartile Range | | 3.70 |  |
|  |  | Skewness | | 2.541 | .794 |
|  |  | Kurtosis | | 6.578 | 1.587 |
|  | 6.00 | Mean | | 13.0833 | 2.36297 |
|  |  | 95% Confidence Interval for Mean | Lower Bound | 7.0091 |  |
|  |  |  | Upper Bound | 19.1575 |  |
|  |  | 5% Trimmed Mean | | 12.7981 |  |
|  |  | Median | | 11.8500 |  |
|  |  | Variance | | 33.502 |  |
|  |  | Std. Deviation | | 5.78806 |  |
|  |  | Minimum | | 7.50 |  |
|  |  | Maximum | | 23.80 |  |
|  |  | Range | | 16.30 |  |
|  |  | Interquartile Range | | 8.05 |  |
|  |  | Skewness | | 1.530 | .845 |
|  |  | Kurtosis | | 2.749 | 1.741 |

**VAR00002**

1= L. Liversidgei

2= L. polygala

3= L. whitei

4= L. speciosum

5= L. scoparium AUST

6= L. scoparium NZ

**Oneway**

| **ANOVA** | | | | | |
| --- | --- | --- | --- | --- | --- |
| VAR00002 | | | | | |
|  | Sum of Squares | df | Mean Square | F | Sig. |
| Between Groups | 125927.445 | 5 | 25185.489 | 10.339 | <.001 |
| Within Groups | 63333.322 | 26 | 2435.897 |  |  |
| Total | 189260.767 | 31 |  |  |  |

| **ANOVA Effect Sizes**^a^ | | | | |
| --- | --- | --- | --- | --- |
|  | | Point Estimate | 95% Confidence Interval | |
|  |  |  | Lower | Upper |
| VAR00002 | Eta-squared | .665 | .331 | .746 |
|  | Epsilon-squared | .601 | .202 | .697 |
|  | Omega-squared Fixed-effect | .593 | .197 | .690 |
|  | Omega-squared Random-effect | .226 | .047 | .308 |
| a. Eta-squared and Epsilon-squared are estimated based on the fixed-effect model. | | | | |

**Post Hoc Tests**

| **Multiple Comparisons** | | | | | | |
| --- | --- | --- | --- | --- | --- | --- |
| Dependent Variable: VAR00002 | | | | | | |
| Tukey HSD | | | | | | |
| (I) VAR00001 | (J) VAR00001 | Mean Difference (I-J) | Std. Error | Sig. | 95% Confidence Interval | |
|  |  |  |  |  | Lower Bound | Upper Bound |
| 1.00 | 2.00 | -102.68333^*^ | 29.88582 | .022 | -194.5062 | -10.8605 |
|  | 3.00 | -4.92000 | 31.21472 | 1.000 | -100.8258 | 90.9858 |
|  | 4.00 | 20.40000 | 36.04365 | .992 | -90.3425 | 131.1425 |
|  | 5.00 | 70.24286 | 28.89922 | .183 | -18.5487 | 159.0344 |
|  | 6.00 | 71.11667 | 29.88582 | .200 | -20.7062 | 162.9395 |
| 2.00 | 1.00 | 102.68333^*^ | 29.88582 | .022 | 10.8605 | 194.5062 |
|  | 3.00 | 97.76333^*^ | 29.88582 | .032 | 5.9405 | 189.5862 |
|  | 4.00 | 123.08333^*^ | 34.89912 | .018 | 15.8574 | 230.3093 |
|  | 5.00 | 172.92619^*^ | 27.45848 | <.001 | 88.5612 | 257.2911 |
|  | 6.00 | 173.80000^*^ | 28.49501 | <.001 | 86.2504 | 261.3496 |
| 3.00 | 1.00 | 4.92000 | 31.21472 | 1.000 | -90.9858 | 100.8258 |
|  | 2.00 | -97.76333^*^ | 29.88582 | .032 | -189.5862 | -5.9405 |
|  | 4.00 | 25.32000 | 36.04365 | .980 | -85.4225 | 136.0625 |
|  | 5.00 | 75.16286 | 28.89922 | .133 | -13.6287 | 163.9544 |
|  | 6.00 | 76.03667 | 29.88582 | .148 | -15.7862 | 167.8595 |
| 4.00 | 1.00 | -20.40000 | 36.04365 | .992 | -131.1425 | 90.3425 |
|  | 2.00 | -123.08333^*^ | 34.89912 | .018 | -230.3093 | -15.8574 |
|  | 3.00 | -25.32000 | 36.04365 | .980 | -136.0625 | 85.4225 |
|  | 5.00 | 49.84286 | 34.05805 | .689 | -54.7990 | 154.4847 |
|  | 6.00 | 50.71667 | 34.89912 | .695 | -56.5093 | 157.9426 |
| 5.00 | 1.00 | -70.24286 | 28.89922 | .183 | -159.0344 | 18.5487 |
|  | 2.00 | -172.92619^*^ | 27.45848 | <.001 | -257.2911 | -88.5612 |
|  | 3.00 | -75.16286 | 28.89922 | .133 | -163.9544 | 13.6287 |
|  | 4.00 | -49.84286 | 34.05805 | .689 | -154.4847 | 54.7990 |
|  | 6.00 | .87381 | 27.45848 | 1.000 | -83.4911 | 85.2388 |
| 6.00 | 1.00 | -71.11667 | 29.88582 | .200 | -162.9395 | 20.7062 |
|  | 2.00 | -173.80000^*^ | 28.49501 | <.001 | -261.3496 | -86.2504 |
|  | 3.00 | -76.03667 | 29.88582 | .148 | -167.8595 | 15.7862 |
|  | 4.00 | -50.71667 | 34.89912 | .695 | -157.9426 | 56.5093 |
|  | 5.00 | -.87381 | 27.45848 | 1.000 | -85.2388 | 83.4911 |
| *. The mean difference is significant at the 0.05 level. | | | | | | |

**Homogeneous Subsets**

| **VAR00002** | | | |
| --- | --- | --- | --- |
| Tukey HSD^a,b^ | | | |
| VAR00001 | N | Subset for alpha = 0.05 | |
|  |  | 1 | 2 |
| 6.00 | 6 | 13.0833 |  |
| 5.00 | 7 | 13.9571 |  |
| 4.00 | 3 | 63.8000 |  |
| 1.00 | 5 | 84.2000 |  |
| 3.00 | 5 | 89.1200 |  |
| 2.00 | 6 |  | 186.8833 |
| Sig. |  | .184 | 1.000 |
| Means for groups in homogeneous subsets are displayed. | | | |
| a. Uses Harmonic Mean Sample Size = 4.961. | | | |
| b. The group sizes are unequal. The harmonic mean of the group sizes is used. Type I error levels are not guaranteed. | | | |
