# Supplementary material for: Aromatic profiles and 15-LOX inhibitory effects of Australian and New Zealand Manuka honeys
Source: PLoS One. 2026 Nov 3;21(11):e0361877. doi: 10.1371/journal.pone.0361877 (PMC13730423; doi:10.1371/journal.pone.0361877)
Supplement: Supplementary file 5 [file pone.0361877.s005.docx]

3-PLA concentrations vs *Leptospermum* species

**Descriptives**

| **Descriptive Statistics** | | | | | |
| --- | --- | --- | --- | --- | --- |
|  | N | Minimum | Maximum | Mean | Std. Deviation |
| VAR00002 | 32 | 126.00 | 1295.80 | 447.1687 | 343.29006 |
| Valid N (listwise) | 32 |  |  |  |  |

**Oneway**

| **ANOVA** | | | | | |
| --- | --- | --- | --- | --- | --- |
| VAR00002 | | | | | |
|  | Sum of Squares | df | Mean Square | F | Sig. |
| Between Groups | 2778070.010 | 5 | 555614.002 | 16.506 | <.001 |
| Within Groups | 875220.078 | 26 | 33662.311 |  |  |
| Total | 3653290.089 | 31 |  |  |  |

| **ANOVA Effect Sizes**^a^ | | | | |
| --- | --- | --- | --- | --- |
|  | | Point Estimate | 95% Confidence Interval | |
|  |  |  | Lower | Upper |
| VAR00002 | Eta-squared | .760 | .494 | .819 |
|  | Epsilon-squared | .714 | .397 | .784 |
|  | Omega-squared Fixed-effect | .708 | .389 | .778 |
|  | Omega-squared Random-effect | .326 | .113 | .412 |
| a. Eta-squared and Epsilon-squared are estimated based on the fixed-effect model. | | | | |

1= L. Liversidgei

2= L. polygala

3= L. whitei

4= L. speciosum

5= L. scoparium AUST

6= L. scoparium NZ

**Post Hoc Tests**

| **Multiple Comparisons** | | | | | | |
| --- | --- | --- | --- | --- | --- | --- |
| Dependent Variable: VAR00002 | | | | | | |
| Tukey HSD | | | | | | |
| (I) VAR00001 | (J) VAR00001 | Mean Difference (I-J) | Std. Error | Sig. | 95% Confidence Interval | |
|  |  |  |  |  | Lower Bound | Upper Bound |
| 1.00 | 2.00 | -180.06000 | 111.09837 | .593 | -521.4047 | 161.2847 |
|  | 3.00 | -155.90000 | 116.03846 | .759 | -512.4229 | 200.6229 |
|  | 4.00 | 6.77333 | 133.98967 | 1.000 | -404.9039 | 418.4506 |
|  | 5.00 | -197.03143 | 107.43074 | .463 | -527.1075 | 133.0447 |
|  | 6.00 | -852.49333^*^ | 111.09837 | <.001 | -1193.8381 | -511.1486 |
| 2.00 | 1.00 | 180.06000 | 111.09837 | .593 | -161.2847 | 521.4047 |
|  | 3.00 | 24.16000 | 111.09837 | 1.000 | -317.1847 | 365.5047 |
|  | 4.00 | 186.83333 | 129.73494 | .703 | -211.7714 | 585.4381 |
|  | 5.00 | -16.97143 | 102.07491 | 1.000 | -330.5920 | 296.6491 |
|  | 6.00 | -672.43333^*^ | 105.92814 | <.001 | -997.8928 | -346.9739 |
| 3.00 | 1.00 | 155.90000 | 116.03846 | .759 | -200.6229 | 512.4229 |
|  | 2.00 | -24.16000 | 111.09837 | 1.000 | -365.5047 | 317.1847 |
|  | 4.00 | 162.67333 | 133.98967 | .826 | -249.0039 | 574.3506 |
|  | 5.00 | -41.13143 | 107.43074 | .999 | -371.2075 | 288.9447 |
|  | 6.00 | -696.59333^*^ | 111.09837 | <.001 | -1037.9381 | -355.2486 |
| 4.00 | 1.00 | -6.77333 | 133.98967 | 1.000 | -418.4506 | 404.9039 |
|  | 2.00 | -186.83333 | 129.73494 | .703 | -585.4381 | 211.7714 |
|  | 3.00 | -162.67333 | 133.98967 | .826 | -574.3506 | 249.0039 |
|  | 5.00 | -203.80476 | 126.60834 | .600 | -592.8032 | 185.1937 |
|  | 6.00 | -859.26667^*^ | 129.73494 | <.001 | -1257.8714 | -460.6619 |
| 5.00 | 1.00 | 197.03143 | 107.43074 | .463 | -133.0447 | 527.1075 |
|  | 2.00 | 16.97143 | 102.07491 | 1.000 | -296.6491 | 330.5920 |
|  | 3.00 | 41.13143 | 107.43074 | .999 | -288.9447 | 371.2075 |
|  | 4.00 | 203.80476 | 126.60834 | .600 | -185.1937 | 592.8032 |
|  | 6.00 | -655.46190^*^ | 102.07491 | <.001 | -969.0825 | -341.8413 |
| 6.00 | 1.00 | 852.49333^*^ | 111.09837 | <.001 | 511.1486 | 1193.8381 |
|  | 2.00 | 672.43333^*^ | 105.92814 | <.001 | 346.9739 | 997.8928 |
|  | 3.00 | 696.59333^*^ | 111.09837 | <.001 | 355.2486 | 1037.9381 |
|  | 4.00 | 859.26667^*^ | 129.73494 | <.001 | 460.6619 | 1257.8714 |
|  | 5.00 | 655.46190^*^ | 102.07491 | <.001 | 341.8413 | 969.0825 |
| *. The mean difference is significant at the 0.05 level. | | | | | | |

**Homogeneous Subsets**

| **VAR00002** | | | |
| --- | --- | --- | --- |
| Tukey HSD^a,b^ | | | |
| VAR00001 | N | Subset for alpha = 0.05 | |
|  |  | 1 | 2 |
| 4.00 | 3 | 179.9667 |  |
| 1.00 | 5 | 186.7400 |  |
| 3.00 | 5 | 342.6400 |  |
| 2.00 | 6 | 366.8000 |  |
| 5.00 | 7 | 383.7714 |  |
| 6.00 | 6 |  | 1039.2333 |
| Sig. |  | .514 | 1.000 |
| Means for groups in homogeneous subsets are displayed. | | | |
| a. Uses Harmonic Mean Sample Size = 4.961. | | | |
| b. The group sizes are unequal. The harmonic mean of the group sizes is used. Type I error levels are not guaranteed. | | | |
