# Supplementary material for: Aromatic profiles and 15-LOX inhibitory effects of Australian and New Zealand Manuka honeys
Source: PLoS One. 2026 Nov 3;21(11):e0361877. doi: 10.1371/journal.pone.0361877 (PMC13730423; doi:10.1371/journal.pone.0361877)
Supplement: Supplementary file 6 [file pone.0361877.s006.docx]

MS concentrations vs *Leptospermum* species

**Descriptives**

[DataSet0]

| **Descriptive Statistics** | | | | | |
| --- | --- | --- | --- | --- | --- |
|  | N | Minimum | Maximum | Mean | Std. Deviation |
| VAR00002 | 32 | 19.30 | 220.90 | 96.3875 | 47.90975 |
| Valid N (listwise) | 32 |  |  |  |  |

**Oneway**

| **ANOVA** | | | | | |
| --- | --- | --- | --- | --- | --- |
| VAR00002 | | | | | |
|  | Sum of Squares | df | Mean Square | F | Sig. |
| Between Groups | 41812.691 | 5 | 8362.538 | 7.410 | <.001 |
| Within Groups | 29342.984 | 26 | 1128.576 |  |  |
| Total | 71155.675 | 31 |  |  |  |

| **ANOVA Effect Sizes**^a^ | | | | |
| --- | --- | --- | --- | --- |
|  | | Point Estimate | 95% Confidence Interval | |
|  |  |  | Lower | Upper |
| VAR00002 | Eta-squared | .588 | .216 | .685 |
|  | Epsilon-squared | .508 | .066 | .625 |
|  | Omega-squared Fixed-effect | .500 | .064 | .618 |
|  | Omega-squared Random-effect | .167 | .013 | .244 |
| a. Eta-squared and Epsilon-squared are estimated based on the fixed-effect model. | | | | |

1= L. Liversidgei

2= L. polygala

3= L. whitei

4= L. speciosum

5= L. scoparium AUST

6= L. scoparium NZ

**Post Hoc Tests**

| **Multiple Comparisons** | | | | | | |
| --- | --- | --- | --- | --- | --- | --- |
| Dependent Variable: VAR00002 | | | | | | |
| Tukey HSD | | | | | | |
| (I) VAR00001 | (J) VAR00001 | Mean Difference (I-J) | Std. Error | Sig. | 95% Confidence Interval | |
|  |  |  |  |  | Lower Bound | Upper Bound |
| 1.00 | 2.00 | -67.89000^*^ | 20.34235 | .028 | -130.3910 | -5.3890 |
|  | 3.00 | -4.32000 | 21.24689 | 1.000 | -69.6001 | 60.9601 |
|  | 4.00 | 57.99333 | 24.53380 | .206 | -17.3857 | 133.3723 |
|  | 5.00 | -2.35429 | 19.67080 | 1.000 | -62.7919 | 58.0834 |
|  | 6.00 | -47.70667 | 20.34235 | .213 | -110.2076 | 14.7943 |
| 2.00 | 1.00 | 67.89000^*^ | 20.34235 | .028 | 5.3890 | 130.3910 |
|  | 3.00 | 63.57000^*^ | 20.34235 | .045 | 1.0690 | 126.0710 |
|  | 4.00 | 125.88333^*^ | 23.75475 | <.001 | 52.8979 | 198.8687 |
|  | 5.00 | 65.53571^*^ | 18.69014 | .019 | 8.1111 | 122.9603 |
|  | 6.00 | 20.18333 | 19.39567 | .900 | -39.4090 | 79.7757 |
| 3.00 | 1.00 | 4.32000 | 21.24689 | 1.000 | -60.9601 | 69.6001 |
|  | 2.00 | -63.57000^*^ | 20.34235 | .045 | -126.0710 | -1.0690 |
|  | 4.00 | 62.31333 | 24.53380 | .149 | -13.0657 | 137.6923 |
|  | 5.00 | 1.96571 | 19.67080 | 1.000 | -58.4719 | 62.4034 |
|  | 6.00 | -43.38667 | 20.34235 | .302 | -105.8876 | 19.1143 |
| 4.00 | 1.00 | -57.99333 | 24.53380 | .206 | -133.3723 | 17.3857 |
|  | 2.00 | -125.88333^*^ | 23.75475 | <.001 | -198.8687 | -52.8979 |
|  | 3.00 | -62.31333 | 24.53380 | .149 | -137.6923 | 13.0657 |
|  | 5.00 | -60.34762 | 23.18226 | .132 | -131.5741 | 10.8788 |
|  | 6.00 | -105.70000^*^ | 23.75475 | .002 | -178.6854 | -32.7146 |
| 5.00 | 1.00 | 2.35429 | 19.67080 | 1.000 | -58.0834 | 62.7919 |
|  | 2.00 | -65.53571^*^ | 18.69014 | .019 | -122.9603 | -8.1111 |
|  | 3.00 | -1.96571 | 19.67080 | 1.000 | -62.4034 | 58.4719 |
|  | 4.00 | 60.34762 | 23.18226 | .132 | -10.8788 | 131.5741 |
|  | 6.00 | -45.35238 | 18.69014 | .184 | -102.7770 | 12.0722 |
| 6.00 | 1.00 | 47.70667 | 20.34235 | .213 | -14.7943 | 110.2076 |
|  | 2.00 | -20.18333 | 19.39567 | .900 | -79.7757 | 39.4090 |
|  | 3.00 | 43.38667 | 20.34235 | .302 | -19.1143 | 105.8876 |
|  | 4.00 | 105.70000^*^ | 23.75475 | .002 | 32.7146 | 178.6854 |
|  | 5.00 | 45.35238 | 18.69014 | .184 | -12.0722 | 102.7770 |
| *. The mean difference is significant at the 0.05 level. | | | | | | |

**Homogeneous Subsets**

| **VAR00002** | | | | |
| --- | --- | --- | --- | --- |
| Tukey HSD^a,b^ | | | | |
| VAR00001 | N | Subset for alpha = 0.05 | | |
|  |  | 1 | 2 | 3 |
| 4.00 | 3 | 20.9667 |  |  |
| 1.00 | 5 | 78.9600 | 78.9600 |  |
| 5.00 | 7 | 81.3143 | 81.3143 | 81.3143 |
| 3.00 | 5 | 83.2800 | 83.2800 | 83.2800 |
| 6.00 | 6 |  | 126.6667 | 126.6667 |
| 2.00 | 6 |  |  | 146.8500 |
| Sig. |  | .069 | .256 | .050 |
| Means for groups in homogeneous subsets are displayed. | | | | |
| a. Uses Harmonic Mean Sample Size = 4.961. | | | | |
| b. The group sizes are unequal. The harmonic mean of the group sizes is used. Type I error levels are not guaranteed. | | | | |
